# Reactive Oxygen Species‐Responsive Nanoparticles Toward Extracellular Matrix Normalization for Pancreatic Fibrosis Regression

**DOI:** 10.1002/advs.202401254

**Published:** 2024-03-14

**Authors:** Liang Qi, Bo‐Wen Duan, Hui Wang, Yan‐Jun Liu, Han Han, Meng‐Meng Han, Lei Xing, Hu‐Lin Jiang, Stephen J. Pandol, Ling Li

**Affiliations:** ^1^ Department of Endocrinology Zhongda Hospital School of Medicine Southeast University Nanjing 210009 China; ^2^ State Key Laboratory of Natural Medicines Department of Pharmaceutics China Pharmaceutical University Nanjing 210009 China; ^3^ Jiangsu Key Laboratory of Druggability of Biopharmaceuticals China Pharmaceutical University Nanjing 210009 China; ^4^ Division of Gastroenterology Department of Medicine Cedars‐Sinai Medical Center Los Angeles CA 90048 USA; ^5^ Basic and Translational Pancreatic Research Cedars‐Sinai Medical Center Los Angeles CA 90048 USA; ^6^ Institute of Glucose and Lipid Metabolism Southeast University Nanjing 210009 China; ^7^ Department of Clinical Science and Research Zhongda Hospital School of Medicine Southeast University Nanjing 210009 China

**Keywords:** collagen cross‐linking, extracellular matrix, Pancreatic fibrosis, pancreatic stellate cells, reactive oxygen species

## Abstract

Pancreatic fibrosis (PF) is primarily characterized by aberrant production and degradation modes of extracellular matrix (ECM) components, resulting from the activation of pancreatic stellate cells (PSCs) and the pathological cross‐linking of ECM mediated by lysyl oxidase (LOX) family members. The excessively deposited ECM increases matrix stiffness, and the over‐accumulated reactive oxygen species (ROS) induces oxidative stress, which further stimulates the continuous activation of PSCs and advancing PF; challenging the strategy toward normalizing ECM homeostasis for the regression of PF. Herein, ROS‐responsive and Vitamin A (VA) decorated micelles (named LR‐SSVA) to reverse the imbalanced ECM homeostasis for ameliorating PF are designed and synthesized. Specifically, LR‐SSVA selectively targets PSCs via VA, thereby effectively delivering siLOXL1 and resveratrol (RES) into the pancreas. The ROS‐responsive released RES inhibits the overproduction of ECM by eliminating ROS and inactivating PSCs, meanwhile, the decreased expression of LOXL1 ameliorates the cross‐linked collagen for easier degradation by collagenase which jointly normalizes ECM homeostasis and alleviates PF. This research shows that LR‐SSVA is a safe and efficient ROS‐response and PSC‐targeted drug‐delivery system for ECM normalization, which will propose an innovative and ideal platform for the reversal of PF.

## Graphical abstract

Nanoparticles named LR‐SSVA selectively target pancreatic stellate cells (PSCs) via Vitamin A, thereby effectively delivering siLOXL1 and resveratrol (RES) into the pancreas. The reactive oxygen species (ROS)‐responsive released RES inhibit the overproduction of extracellular matrix (ECM) by eliminating ROS and inactivating PSCs, meanwhile, the decreased expression of LOXL1 ameliorates the cross‐linked collagen for easier degradation by collagenase, which jointly normalize ECM homeostasis and alleviate pancreatic fibrosis.

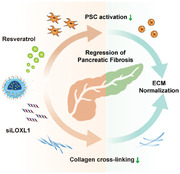

## 1 Introduction

Pancreatic fibrosis (PF) is the main pathological feature of chronic pancreatitis (CP), which is attributed to progressive inflammation and continuous wound‐healing response of pancreas.^[^ ^1^ ^]^ It is characterized by excessively deposited extracellular matrix (ECM) components caused by overproduction and hindered degradation of ECM proteins such as collagen (type I and type III) and fibronectin.^[^ ^2^ ^]^ The abnormal cross‐linking and accumulation of ECM drive PF to stiffen the matrix and promote malignancy in the clinical absence of effective pharmacological intervention.^[^ ^3^ ^]^ Normalizing the production and degradation mode of ECM proteins, and restoring its physiological homeostasis may present a promising strategy for resolving PF.

Accumulating evidence indicates that activated pancreatic stellate cells (PSCs) play a pivotal role in the progression of PF. In response to persistent inflammatory stimulation and external injury of the pancreas, a large number of cytokines, and inflammatory mediators including TGF‐β, TNF‐α, and IL‐1β are secreted by acinar cells and macrophages, which leads to the activation of PSCs.^[^ ^4^ ^]^ The activated PSCs proliferate, migrate, and actively secrete excessive ECM proteins.^[^ ^5^ ^]^ Additionally, covalent cross‐linking among intra‐ and intermolecular protein chains of ECM proteins, prominently mediated by lysyl oxidase (LOX) family members, leads to the stabilization and weakening of ECM turnover.^[^ ^6^ ^]^ Especially, the pathologically cross‐linked ECM provides collagenase resistance to collagen, and renders its degradation difficult. Moreover, the excessive deposition of ECM increases matrix stiffness and further stimulates the continuous activation of PSCs, ultimately promoting PF progression.^[^ ^7^ ^]^

LOX and its family members, lysyl oxidase‐like (LOXL 1–4) are copper‐dependent amino oxidases, which are responsible for catalyzing structural ECM cross‐linking in fibrotic organs.^[^ ^8^ ^]^ Several members of LOX family have been found excessively secreted in progressive fibrotic tissues by activated primary source cells such as fibroblast and stellate cells.^[^ ^9^ ^]^ Previous studies have extensively documented the up‐regulation of certain LOX family members, particularly LOX and LOXL1‐2 in general fibrosis.^[^ ^8^, ^10^ ^]^ The increased activity of LOX family members helps render fibrotic tissues less reversible and creates a stiffer matrix, which leads to vicious reciprocation by further activation of fibrogenic effector cells.^[^ ^11^ ^]^ To date, targeted strategies specifically aimed at inhibiting LOX family members using small‐molecule inhibitors or small interfering RNA (siRNAs) have shown therapeutic potential for fibrosis of multiple organs.^[^ ^10^, ^12^ ^]^ Previous research has verified the effect in ECM normalization by suppressing LOX activity,^[^ ^13^ ^]^ and LOXL1‐targeted interventions have shown promising therapeutic effects in advancing fibrosis,^[^ ^8^, ^14^ ^]^ suggesting LOX family members as attractive drug targets, especially in combination with conventional antifibrotic agents.^[^ ^15^ ^]^

Furthermore, oxidative stress and elevated reactive oxygen species (ROS) are the driving force for pressure‐induced PSCs activation and ECM synthesis. The accumulation of cross‐linked ECM proteins leads to increased matrix stiffness, resulting in higher localized pressure within fibrotic pancreatic tissue compared to normal tissue. Additionally, heightened ROS levels contribute to PF progression and damage pancreatic parenchymal cells by activating downstream redox‐sensitive signaling pathways.^[^ ^16^ ^]^ Antioxidants are currently recognized as effective antifibrotic agents for PF due to their anti‐oxidative and anti‐inflammatory properties.^[^ ^17^ ^]^ As a natural polyphenol, resveratrol (RES) has been verified to inhibit the activation, invasion, migration, and glycolysis of PSCs.^[^ ^17^, ^18^ ^]^ Combining targeted inhibition of LOX family members with RES‐induced ROS elimination represents a promising strategy for reversing PF.

**Figure 1:**
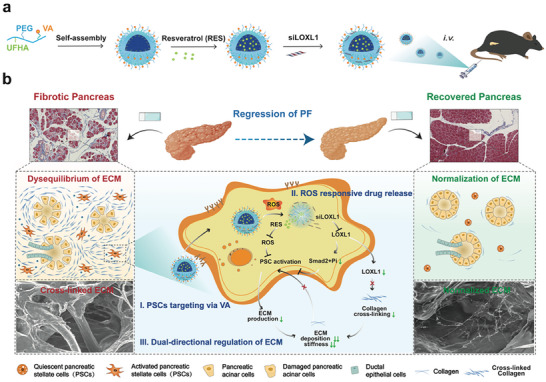
Schematic illustration of the preparation and expected mechanism of LR‐SSVA toward ECM normalization for the regression of PF.

However, due to the challenges in pancreas‐targeted delivery of therapeutic genes and chemical drugs, it is urgent to develop a PSC‐targeted drug delivery platform for the therapy of PF specifically. Inspired by our previous efforts,^[^ ^15^, ^19^ ^]^ we identified significant differentially expressed genes of LOX family members in PF, and designed ROS‐responsive cationic polymer micelles for drugs delivery. The polymer micelles were decorated with vitamin A (VA) for PSC‐targeting, wrapped RES in, and adsorbed RNA interference drug (siLOXL1) onto the surface; the whole system was termed LR‐SSVA. When LR‐SSVA was injected into mice via the tail vein, VA facilitated targeted absorption by PSCs, with drug release triggered by ROS‐sensitive components. RES reduced ROS levels and inhibited PSCs activation; siLOXL1 significantly decreased LOXL1 expression in PSCs, thereby suppressing collagen cross‐linking and inhibiting small mother against decapentaplegic (Smad) phosphorylation mediated through mechanotransduction pathways (**Figure** **1**). This approach specifically addresses fibrogenic cell inactivation and ECM homeostasis restoration, which will effectively reverse PF and provide an innovative therapeutic strategy for CP.

## 2 Results and Discussion

### 2.1 Expression of LOX Family Members in Healthy and Fibrotic Pancreas

**Figure 2:**
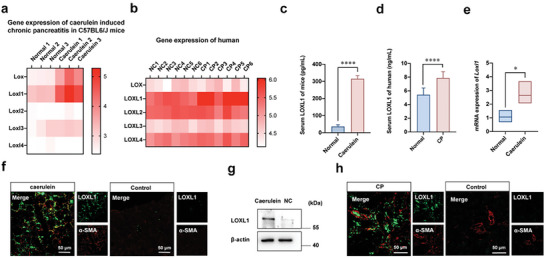
Expression patterns of LOX family members in CP. a,b) RNA sequencing data in the CNBI database of caerulein‐induced CP mice (GSE 41 418) and tissues from human CP patients (GSE 143 754). c,d) ELISA analysis of serum LOXL1 expressions of caerulein‐induced CP mice and CP patients. e,g) Western blot and qPCR analysis for proteins and mRNA levels of caerulein‐induced CP mice. f,h) Immunofluorescence staining of LOXL1 (green) and α‐SMA (red) in caerulein‐induced CP mice and CP patients, respectively. Values are expressed as means ± SD (^*^ *P* < 0.05, ^****^ *P* < 0.0001).

The RNA sequencing data in the CNBI database of caerulein‐induced CP mice (GSE 41 418) and tissues from human CP patients (GSE 143 754) were analyzed, to screen the expression patterns of LOX and LOXL1‐4. It showed that the expression of LOXL1 was notably up‐regulated in both mouse and human fibrotic pancreas (**Figure** **2**a,b). Although the expression of LOXL4 in the CP patients was slightly higher than that in the control group, the difference was not statistically significant. The caerulein‐induced CP models were established according to the standard procedure as described in the experimental sections, and the pancreas samples were harvested to obtain proteins and RNA. The serum concentration of LOXL1 in fibrotic mice and CP patients were all detected to be increased than that in the normal group (Figure 2c,d). Western blot and qPCR analysis showed significantly increased mRNA and protein expressions of LOXL1 in pancreas of fibrotic mice (Figure 2e,g). Additionally, immunofluorescence staining indicated that the expressions of LOXL1 (green) and α‐SMA (red) were notably enhanced in PF mice, and merged into yellow fluorescence as shown in Figure 2f. Furthermore, the pancreas tissue of CP patients was acquired to verify the preceding findings. The pancreatic LOXL1 expression of CP patients were higher than that of healthy participants, and the immunofluorescence staining of LOXL1 and α‐SMA exhibited a similar tendency to pancreas of fibrotic mice (Figure 2h).

LOX family members are responsible for the cross‐linking of ECM fibers, which promoting the progression of PF and making it difficult to alleviate. As the most remarkable increased member of LOX family, the targeted intervention of LOXL1 might be effective strategy for the regression of PF. Therefore, siRNA was applied to inhibit LOXL1 expression, and the best‐interfering sequence was selected referring to Western blot and qPCR analysis (Figure S1, Supporting Information).

### 2.2 Synthesis and Characterization of LR‐SSVA

**Figure 3:**
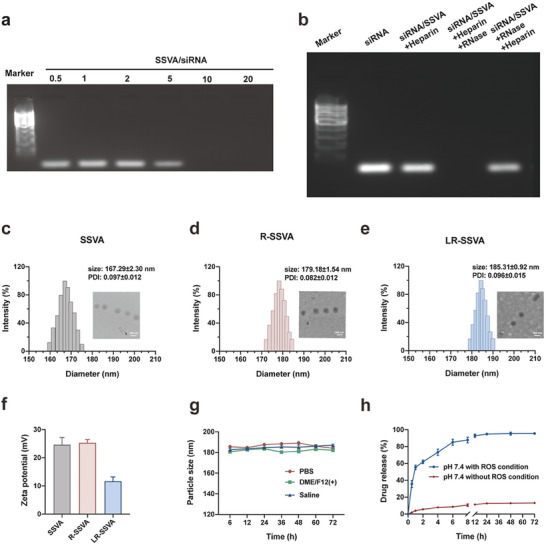
Synthesis and characterization of different preparations. a,b) Gene adsorption capacity and degradation resistance from RNase of SSVA. c–e) Average size distributions of SSVA, R‐SSVA, and LR‐SSVA as measured by DLS, and representative TEM images. Scale bar: 200 nm. f) Zeta potentials of SSVA, R‐SSVA and LR‐SSVA. g) Stability of LR‐SSVA in PBS, saline, and DME/F12 with 10% FBS over 72 h. h) Release kinetics of RES within 72 h, with/without ROS condition.

The synthetic process of LR‐SSVA was shown in Figure S2 (Supporting Information) and the ^1^H‐NMR analysis was exhibited in Figures S3–S8 (Supporting Information). The average particle size of SSVA was determined to be 167.29 ± 2.30 nm, which increased to 179.18 ± 1.54 nm when wrapped with RES and further to 185.31 ± 0.92 nm after gene adsorbed (**Figure** **3**c–e). TEM images showed the spherical structures of different formulations (Figure 3c–e). The values of zeta potential were measured to be 24.67 ± 2.05, 25.33 ± 0.94, and 11.67 ± 1.25 mV (Figure 3f), respectively. The positive charge of SSVA makes it possible to adsorb negatively charged siLOXL1 through electrostatic force.

As **Figure** **3**a showed, siRNA was partially adsorbed when the mass ratio of SSVA/siRNA was 5, and completely adsorbed when the mass ratio reached 10. As gene drugs can be degraded by abundant Ribonuclease (RNase) in blood, effectively protecting gene from degradation could greatly improve the therapeutic effect. According to the results in Figure 3b, siRNA adsorbed on micelles was replaced when heparin sodium was added, showing a similar fluorescence band with the positive control. When treated with heparin sodium and RNase successively, the displaced siRNA can be degraded by RNase; while in the group treated with RNase first and then with heparin sodium, obvious fluorescence bands can be seen as before. The above results indicated that SSVA can protect siRNA from RNase degradation and provide a potential platform for effective delivery of gene drugs. Ultraviolet‐visible spectra of RES were shown in Figure S9a (Supporting Information); the maximum absorption peaks of which was noticed at 306 nm. The concentrations of RES were determined and calculated according to the corresponding standard curves of RES (Figure S9b, Supporting Information). The drug vehicle ratio of 7.5: 0.5 was selected as the optimal prescription as it demonstrated both higher EE% and DLE%, which measured to be 83.67 ± 1.25% and 5.77 ± 0.62% (Table S1, Supporting Information). The EE% and DLE% of different preparations were shown in Table S2 (Supporting Information). LR‐SSVA exhibited satisfactory stability of particle sizes in PBS, saline, and DME/F12 with 10% FBS over 72 h (Figure 3g).

DCFH can be oxidized to DCF with green fluorescence, which was applied to detect the level of cellular ROS. The non‐ROS‐responsive micelles (SVA) without thioketal units were identified as positive control. The thioketal unit in SSVA can react with ROS, thus reducing ROS and weakening its stimulation to PSCs. The decreased green fluorescence in LR‐SSVA proves that ROS had been effectively consumed (Figure S10, Supporting Information). The release kinetics of LR‐SSVA within 72 h, with/without ROS environment, were measured following the protocols described as the experimental sections. RES loaded in SSVA only released 13.22% in PBS (pH 7.4) for 72 h, nevertheless, 85% of RES was released in 6 h in the presence of ROS owing to the ROS response unit of SSVA (Figure 3h). SSVA can be destroyed by ROS and make the originally tight micelles loose and porous, which leads to the rapid release of RES.

### 2.3 Resolution of PSCs Activation and ECM Deposition In Vitro

**Figure 4:**
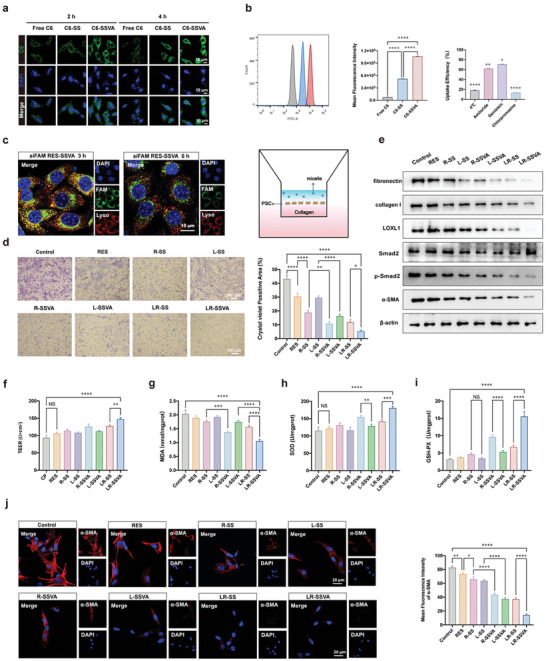
Cellular pharmacodynamics of micelles. a,b) The cellular uptake ability of free C6, C6‐SS, C6‐SSVA, and uptake mechanism of C6‐SSVA. c) The lysosomal escape capacity of siFAM/RES‐SSVA. d) Invasion assay of mPSCs after different treatments, the results were semi‐quantified by image J. Scale bar: 100 µm. e) Western blot analysis of α‐SMA, p‐Smad2, Smad2, LOXL1, collagen I, fibronectin and β‐actin (n = 3). (f) TEER values of mPSCs. g–i) MDA, SOD, and GSH‐PX activities of mPSCs treated with different preparations. j) Immunofluorescence staining of α‐SMA of mPSCs treated with various preparations. Values are expressed as means ± SD (^*^ *P* < 0.05, ^**^ *P* < 0.01, ^***^ *P* < 0.001, ^****^ *P* < 0.0001, NS, no significant difference; n = 3).

Effective cellular uptake and lysosomal escape of micelles are the core links for efficient drug targeted‐delivery. The cellular uptake ability of C6‐labeled micelles (C6‐SSVA) was investigated by CLSM at 2 h and 4 h after being treated with PSCs derived from mice (mPSCs). Highest intensity of C6 signals (green) was observed in mPSCs after incubating with C6‐SSVA for 2 h, indicating its enhanced cellular uptake ability due to the binding of VA. When extending the incubation time to 4 h, the intracellular C6 signals of C6‐SS and C6‐SSVA were improved, suggesting a time‐dependent manner of cellular uptake (**Figure** **4**a). Meanwhile, flow cytometry was used for the quantitatively evaluation of fluorescence intensity after 4 h, which further confirmed a twofold increase in the internalization of C6‐SSVA compared to the C6‐SS group (Figure 4b). Additionally, the uptake mechanism of SSVA is further studied by using amiloride hydrochloride, genistein hydrochloride, and chlorpromazine hydrochloride as macrocytosis inhibitor, inhibitors of caveolin and clathrin‐mediated endocytosis respectively. As Figure 4b shows, the uptake ability of SSVA can be significantly inhibited under 4°C, suggesting the energy‐dependent endocytosis of SSVA. It decreased to 62.13%, 71.0%, and 13.67% of the original cellular uptake ability after intervention by amiloride, genistein, and chlorpromazine, demonstrating multiple endocytosis pathways mediated by energy‐dependent macrocytosis, caveolin, and clathrin. The lysosome escape ability of C6‐SSVA was evaluated at 3 h and 6 h after being treated with mPSCs. Green fluorescence of C6 and red of lysosome highly overlapped at 3 h and separated after 6 h, indicating the escape of C6‐SSVA from the lysosome (Figure 4c).

Subsequently, we investigated the migration and invasion ability of PSCs In vitro. Initially, the invasion abilities of mPSCs treated with different preparations were measured via a Transwell device. Crystal violet staining showed plenty of mPSCs outside the chamber, suggesting active invasion abilities of activated mPSCs; which gradually decreased after treated, and the LR‐SSVA group exhibited lowest amounts of mPSCs (Figure 4d). TEER between apical and basal compartment of the Transwell was detected to evaluate the integrity of mPSCs. The higher TEER value suggests a tight connection of cells, and increased migration of mPSCs resulting in decreased TEER values (Figure 4f). Thereafter, the activation of PSCs was evaluated by Western blot analysis and immunofluorescence staining. It is revealed that as compared to the control group, the expression of smooth muscle alpha‐actin (α‐SMA) was decreased after treatments (Figure 4j). Furthermore, Western blot exhibited the increased expressions of fibronectin, collagen I, α‐SMA, LOXL1, and phosphorylated Smad 2 in activated mPSCs, which evidently reduced after treatments (Figure 4e). These aforementioned experimental results indicated that the delivery of siLOXL1 successfully inhibited the expression of LOXL1 In vitro, and the markers of PSC activation and ECM deposition were decreased after the treatment of LR‐SSVA. Moreover, the activities of antioxidant enzymes (SOD and GSH‐PX) and the concentration of lipid peroxidation product (MDA) were measured. The decreased serum activities of SOD and GSH‐PX as well as elevated MDA activity were detected in CP mice, which could be reversed after intervention. The treatment with LR‐SSVA could effectively cope with ROS and oxidative damage (Figure 4g–i).

### 2.4 PSC‐Targeted Aggregation Ability of LR‐SSVA

**Figure 5:**
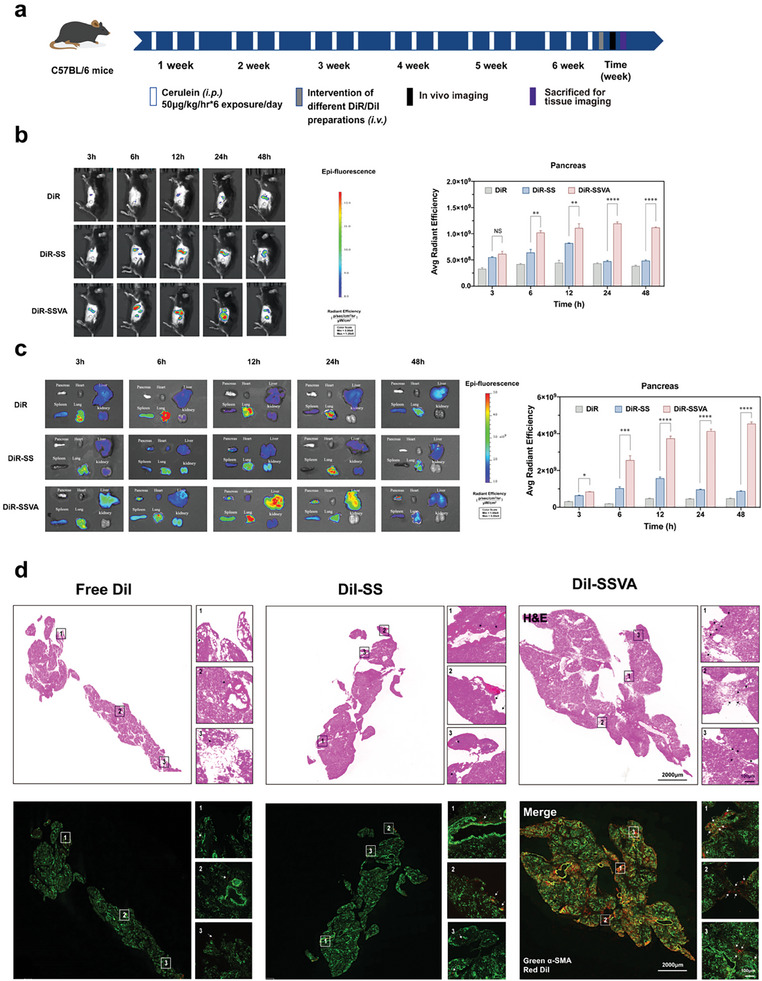
DiR/DiI‐labeled micelles distributions in mice of PF. a) schematic diagram of animal studies. b) Representative near‐infrared fluorescent images In vivo of fibrotic mice, acquired at 3, 6, 12, 24, and 48 h after tail vein injection of different formulations. c) Fluorescence intensity of major organs (pancreas, heart, lung, liver, kidney, and spleen) of PF mice, expressed as average radiant efficiency units. d) HE and immunofluorescence staining images of pancreatic tissues in different groups. Colocalization of free DiI and DiI‐labeled micelles with α‐SMA in fibrotic pancreas are shown in the magnified images. DiI (red), α‐SMA (green). Scale bars: panoramic image/2000 µm and magnified image/100 µm. Arrows indicate DiI fluorescence. Values are expressed as means ± SD (^*^ *P* < 0.05, ^**^ *P* < 0.01, ^***^ *P* < 0.001, ^****^ *P* < 0.0001, NS, no significant difference; n = 3).

Animal models of PF were established by intraperitoneal injection with caerulein into C57BL/6 male mice for 6 weeks (**Figure** **5**a). A fluorescence probe (DiR) was applied to assess the tissue distribution of micelles. After modeling, free DiR and DiR‐labeled preparations (DiR‐SS and DiR‐SSVA) were injected separately for In vivo imaging. Representative near‐infrared fluorescence images of different groups of mice were acquired at 3, 6, 12, 24, and 48 h, respectively. Additionally, the main organs (heart, lung, liver, kidney and spleen) were collected to detect the fluorescence intensities with excitation wavelengths of 745 nm and emission wavelengths of 800 nm. As shown in Figure 5b,c, there was hardly any pancreatic accumulation in free DiR group within 48 h, and a small amount of fluorescent signal was observed in the DiR‐SS group within 6–12 h; the average radiant efficiency of DiR‐SSVA was identifiable within 6 h in the pancreas and kept evident till 48 h. Due to the liver is particularly enriched with hepatic stellate cells (HSCs), it is worth noting that HSCs also exhibit attractions toward VA,^[^ ^20^ ^]^ resulting in a certain degree of DiR fluorescence within the liver for the DiR‐SSVA group. Although inevitable, this phenomenon does not cause a significant decrease of DiR accumulation within pancreatic tissue. It had been demonstrated that the caerulein‐conducted mouse showed certain degree of pulmonary injury,^[^ ^21^ ^]^ pathological injuries such as lung inflammation led to increased permeability of capillaries and recruitment of large numbers of mononuclear macrophages, thus increasing the accumulation of long‐circulating nanoparticles in the lung (Figure 5c). Therefore, despite the presence of a minor off‐target effect in the VA targeting strategy, it does not significantly impede its pancreatic‐specific accumulation; instead, there is a time‐dependent increase in its accumulation specifically within the pancreas.

In order to further verify the targeting ability of SSVA, various formulations labeled by DiI (DiI‐SS and DiI‐SSVA) were injected for three consecutive days into the fibrotic mice, for immunofluorescence staining and H&E staining of consecutive frozen sections. The α‐SMA (green) and DiI (red) expressions were observed both in panoramic and magnified images (Figure 5d). In the free DiI and DiI‐SS group, only a limited amount of red fluorescence was observed surrounding the pancreatic vessels and at the periphery of the pancreatic tissue. While, the DiI‐SSVA group exhibited the most intense red fluorescence, and it was evident that the α‐SMA‐positive area coincided with the DiI‐positive region within the interstitial space (as shown by black arrows in HE staining and white arrows in immunofluorescence staining); which further substantiating SSVA's targeting efficacy toward PSCs. The above experiments suggest that DiI‐SSVA could target PSCs and increase pancreatic accumulation with the help of VA.

### 2.5 Antifibrotic Effects of LR‐SSVA In Vivo

**Figure 6:**
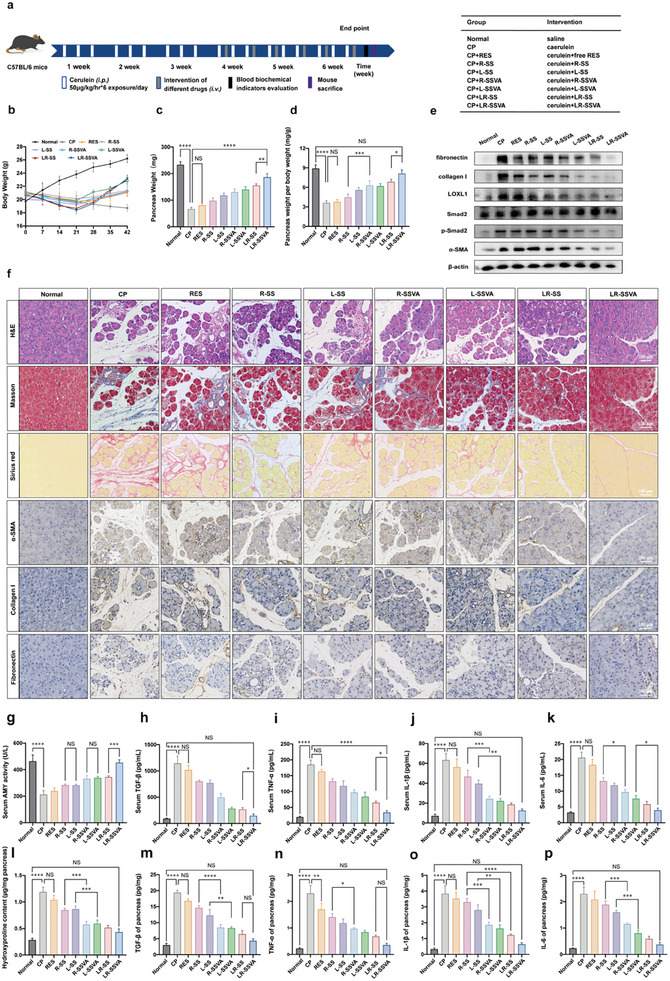
Pharmacodynamics of micelles In vivo. a) Schematic diagram of animal studies. b) Body weight of healthy mice and PF mice before and after treated with different preparations over 6 weeks. c,d) Pancreas weight and values of pancreas weight per body weight after the final injection. e) Western blot analysis. f) Representative images of pancreatic HE, Sirius Red, Masson, and IHC staining (collagen I, fibronectin, and α‐SMA,). Scale bars: 100 µm (200x). g) Serum AMY activity in healthy and PF mice with different treatments. l) Hydroxyproline contents of the pancreas in healthy and PF mice in different groups. h–k,m–p) ELISA assay for the serum and pancreatic concentrations of TGF‐β, TNF‐α, IL‐1β, and IL‐6. Values are expressed as means ± SD (^*^ *P* < 0.05, ^**^ *P* < 0.01, ^***^ *P* < 0.001, ^****^ *P* < 0.0001, NS, no significant difference; n = 3).

Subsequently, the antifibrotic ability of LR‐SSVA was assessed. From the fourth week of modeling, free RES and different preparations (R‐SS, L‐SS, R‐SSVA, L‐SSVA, LR‐SS, and LR‐SSVA) were administrated via tail vein twice a week for three weeks, and mice were dissected to collect main organs for follow‐up biochemical and immunohistological analysis (**Figure** **6**a). The images of pancreatic tissues were obtained and exhibited in Figure S11 (Supporting Information). A stiffer and smaller pancreas was observed in fibrotic mice, which demonstrated different extents of morphological recovery with treatments. Body weights of all the laboratory mice were monitored over the whole experiment, which continuously decreased in the CP group resulting from recurrent pancreatitis induced by the stimulation of caerulein. However, those groups showed varying degrees of weight recovery after different interventions. The healthy control group treated with PBS (negative control) exhibited continuously increased body weight during 6 weeks’ of regular feeding (Figure 6b). Afterward, pancreatic tissues were collected and weighed, which were further calculated as pancreas weight per body weight (Figure 6c,d). The fibrotic pancreas was significantly lighter than healthy pancreas, and the weight of pancreatic tissues gradually gained with fibrosis amelioration. The ratio of pancreas weight to body weight showed the same tendency.

Our previous studies had confirmed the antifibrotic ability by ECM regulation in various organ fibrosis.^[^ ^15^, ^19^, ^22^ ^]^ To further analyze the regression of PF, tissue sections of different groups were stained to assess ECM deposition. From H&E staining of fibrotic mice, the acinar architecture was severely destroyed with inflammatory cells accumulated, and ECM proteins were excessively deposited. Recovered acinar units and reduced ECM were observed after treatment, especially with LR‐SSVA (Figure 6f). Sirius Red staining and Masson's trichrome staining were combined and applied for analyzing collagen deposition. Only perivascular collagen fibers in normal pancreas were stained in blue or red by Masson or Sirius Red staining. Nevertheless, full view of red‐ or blue‐stained areas were shown in pancreas of fibrotic mice. After being treated with various preparations, deposited collagen was ameliorated to different degrees (Figure 6f; Figure S13, Supporting Information). Immunohistochemical staining of collagen I, fibronectin, and α‐SMA revealed analogous tendencies, suggesting the PSCs activation and ECM deposition in fibrotic mice, and the PSCs activity was inhibited with ECM ablating after treatments (Figure 6f; Figure S13, Supporting Information). Additionally, the AMY activity and hydroxyproline contents of pancreatic tissues were determined in different groups. The activity of AMY decreased in CP mice due to acinar cells dysfunction, which was improved with the recovery of PF (Figure 6g). Hydroxyproline contents were immensely improved in fibrotic pancreas and reduced after therapy (Figure 6l). Serum and pancreatic inflammatory or fibrotic‐related cytokines, including interleukin‐6 (IL‐6), interleukin‐1β (IL‐1β), transforming growth factor‐β (TGF‐β), and tumor necrosis factor‐α (TNF‐α) were determined to be significantly increased in fibrotic mice and gradually reduced to normal with treatments (Figure 6h–k,m–p). With the progression of PF, the expressions of IL‐1β, IL‐6 TGF‐β, and TNF‐α increased with the activation of PSCs, and massive collagen deposited. Treatment with LR‐SSVA decreased the secretion of cytokines, inhibited the activation of PSCs, and normalized the production and degradation manners of ECM.

The number of white blood cells (WBC), neutrophils (granulocyte ^#^), lymphocytes (Lymph^#^), monocyte (Mon^#^), hemoglobin (HGB), and red blood cells (RBC) in the whole blood of mice were determined after treated (Figure S12, Supporting Information). In the process of CP, inflammatory reaction recruited quantities of inflammatory cells and immune cells, which results in a significant increase in the number of WBC, Lymph^#^, Gran^#^, and Mon^#^ in the blood; reflecting the systemic inflammatory reaction and immune response. After treatments, inflammatory cells and immune cells decreased, which confirmed the alleviation of inflammation. No significant differences were observed in the concentrations of RBC and HGB among the groups.

Except for effectiveness, the biocompatibility, acute and short‐term toxicity of LR‐SSVA were assessed. Initially, the cytotoxicity of SS, SSVA, RES, R‐SS, and R‐SSVA to 266‐6 cells and mice/human PSCs was evaluated within 24 h by methyl thiazolyl tetrazolium (MTT) test. After incubating with the empty micelles for 24 h with a concentration below 100 µg mL^−1^, all three cell types showed high viability, suggesting good safety in vitro of nanosystem (Figure S14, Supporting Information). The acute and short‐term toxicity of LR‐SSVA were investigated by histological analysis, as well as serum testing for hepatotoxicity and nephrotoxicity markers. As Figure S15 (Supporting Information) showed, there was no obvious pathomorphology in the heart, lung, liver, kidney, and spleen from H&E staining; and no significant differences were observed in serum blood urea nitrogen (BUN), creatinine (Cr), alanine aminotransferase (ALT), aspartate aminotransferase (AST) after treated with LR‐SSVA, compared to normal pancreas (Figure S15, Supporting Information). At the end of the pharmacodynamic experiment, fibrotic mice were sacrificed for major organs. Referring to the H&E staining, mild pulmonary injury with thickened alveolar walls of lung lesions, inflammatory cell infiltration, and edema were observed in caerulein‐conducted mice; which disappeared after being treated with LR‐SSVA. No obvious pathmorphology was observed in other major organs in groups with various treatments (Figure S16f, Supporting Information). The serum ALT and AST levels were determined and showed no significant difference among various groups (Figure S16a,b, Supporting Information). The concentrations of BUN and Cr were decreased in fibrotic mice, which recovered to normal levels after treatments (Figure S16c,d, Supporting Information). The blood compatibility assay showed no hemolysis after treated with different concentrations of LR‐SSVA (5–200 µg mL^−1^) (Figure S16e, Supporting Information).

### 2.6 Normalization of ROS and ECM Homeostasis In Vivo

**Figure 7:**
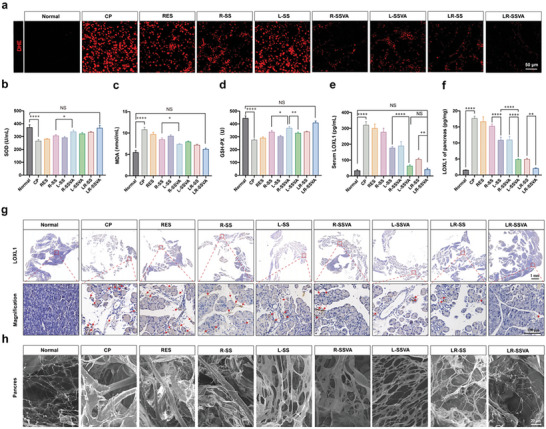
Elimination of ROS and suppression of cross‐linked collagen. a) DHE staining of ROS in healthy and PF mice after different treatments. b–d) Serum activities of SOD, MDA, and GSH‐PX of healthy and PF mice after different treatments. e,f) Serum and pancreatic expressions of LOXL1 in healthy and PF mice after different treatments. g) IHC staining of LOXL1 in different groups. h) Microstructure of the collagen mesh imaged by SEM, in healthy pancreas and fibrotic pancreas with/without treatments. Values are expressed as means ± SD (^*^ *P* < 0.05, ^**^ *P* < 0.01, ^****^ *P* < 0.0001, NS, no significant difference; n = 3).

Furthermore, DHE was used to detect ROS production in frozen tissue sections of pancreas. The CP group showed extremely strong red fluorescence compared with the control group, indicating significantly increased ROS accumulation after modeling. Treatment of LR‐SSVA effectively reduced the production of ROS benefiting from the efficient delivery of RES via SSVA (**Figure** **7**a; Figure S17, Supporting Information). Simultaneously, serum activities of antioxidant enzymes (GSH‐PX and SOD) were decreased obviously, and MDA was increased in the CP group (Figure 7b–d). The aforementioned results demonstrated that the fibrotic mice were in a state of high oxidative stress, while the oxidative damage of modeling mice was completely subsided with the help of LR‐SSVA.

More importantly, we continued to explore the serum and pancreatic LOXL1 expressions by ELISA analysis (Figure 7e,f) as well as immunohistochemistry (Figure 7g). The abovementioned experiments confirmed the decreased concentrations of LOXL1 after being treated with LR‐SSVA, confirming the successful delivery and excellent interfering effectiveness of siLOXL1. To visualize the microstructure of the collagen mesh, healthy pancreas, and fibrotic pancreas with/without treatment were decellularized, and ECM components were imaged by SEM (Figure 7h). The collagen fibers were thin and loosely arranged in the healthy pancreatic tissue, however in fibrotic pancreas, the collagen fibers were heavily crosslinked and thickened to be hardily degraded. The inhibition of LOXL1 could help to suppress collagen cross‐linking and normalize the production and degradation of ECM proteins, which ultimately promotes the regression of PF.

## 3 Conclusion

ECM overproduction and excessive deposition caused by activated PSCs are the prominent characteristics of PF, and elevated ROS jointly drive pressure‐induced PSC activation and ECM synthesis. Pathologically cross‐linked ECM leads to the stabilization and weakening of ECM turnover, which further stimulates continuous activation of PSCs. Herein, we investigated the expressions of LOX family members in PF, and identified LOXL1 as a potential intervention target. Based on these findings, we further designed and synthesized a ROS‐responsive micelles (LR‐SSVA) for the regression of PF. LR‐SSVA demonstrated excellent safety, biocompatibility, and preferential accumulation in the pancreas after tail vein injection due to its PSC‐targeting property mediated by VA. Importantly, the expression of LOXL1 was decreased and ROS were eliminated in fibrotic mice after treatment, thus PSCs were inactivated to normalize the production and degradation of ECM proteins. Therefore, based on the above results, we recommend LOXL1 as an effective intervention target and propose LR‐SSVA as an ideal PSC‐targeted regulatory system to normalize ECM homeostasis for the reversal of PF.

## 4 Experimental Section

### Materials

Trifluoroacetic anhydride, 2‐aminoethane‐1‐mercaptan hydrochloride, 2‐methoxypropylene, oluene‐p‐sulfonic acid, acryloyl chloride, acryloyl chloride, undecfluorohexylamine, cholamine, dicarbonyl imidazole, retinol, and RES were purchased from Aladdin Co., Ltd (Shanghai, China). The hydroxyproline, glutathione‐peroxidase (GSH‐PX), superoxide dismutase (SOD), and malondialdehyde (MDA) assay kit were obtained from Nanjing Jiancheng Bioengineering Institute. 4,6‐diamino‐2‐phenylindole (DAPI), Lyso‐Tracker Red, and AF647‐conjugated Goat Anti‐Rabbit IgG were purchased from Beyotime Biotechnology Co. Ltd. (Shanghai, China). Other chemical reagents were purchased from Nanjing Chemical Reagents Co., Ltd. Total protein extraction kits were purchased from KeyGEN BioTECH Co. Ltd. (Nanjing, China). Confocal dishes were purchased from NEST Biotechnology Co.Ltd. (Wuxi, China). The total RNA isolation kit was purchased from Chengdu Foregene Company Limited. Complementary DNA was obtained with All‐In‐One 5X RT MasterMix from Applied Biological Materials (Nanjing, China). Real‐time fluorescence quantitative polymerase chain reaction (qPCR) was conducted by the 2 × S6 Universal SYBR qPCR Mix (EnzyArtisan Biotech Co., Ltd, China). The siRNA fragments were customized from GenePharma Co., Ltd (Suzhou, China). The Mouse Anti‐Rabbit IgG Antibodies were purchased from GenScript Corporation.

### Ethical Standards

All of the animal experiments were conducted under the protocols approved by the Ministry of Health of the People's Republic of China and followed the Guidelines for the Care and Use of Laboratory Animals of China Pharmaceutical University. All human and animal studies have been performed in accordance with the ethical standards laid down in the 1964 Declaration of Helsinki and its later amendments, and have therefore been approved by the local Ethics Committee (Ethics number: 2018ZDSYLL070‐P01), and registered in the Chinese Clinical Trial Registry (No: ChiCTR1800018247). All persons gave their informed consent prior to their inclusion in the study.

## Conflict of Interest

The authors declare no conflict of interest.

## Author Contributions

L.Q. and B.‐W.D. contributed equally to this work. L.Q., B.‐W.D., H.‐L.J., S.J.P., and L.L. initiated, designed, planned, and oversaw all aspects of the study. L.Q., B.‐W.D., H.W., Y.‐J.L., H.H., M.‐M.H., and L.X. performed the experimental and statistical work, and L.Q. drafted the manuscript. All authors reviewed and edited the final version of the manuscript.

## Supporting information

Supporting Information

## Data Availability

The data that support the findings of this study are available from the corresponding author upon reasonable request.
