# Supplementary material for: Reactive Oxygen Species‐Responsive Nanoparticles Toward Extracellular Matrix Normalization for Pancreatic Fibrosis Regression
Source: Adv Sci (Weinh). 2024 Mar 14;11(19):2401254. doi: 10.1002/advs.202401254 (PMC11109658; doi:10.1002/advs.202401254)
Supplement: Supplementary file 1 — Supporting Information [file ADVS-11-2401254-s001.pdf]

ADVANCED SCIENCE
Open Access

Supporting Information

for *Adv. Sci.*, DOI 10.1002/advs.202401254

Reactive Oxygen Species-Responsive Nanoparticles Toward Extracellular Matrix Normalization for Pancreatic Fibrosis Regression

Liang Qi, Bo-Wen Duan, Hui Wang, Yan-Jun Liu, Han Han, Meng-Meng Han, Lei Xing, Hu-Lin Jiang, Stephen J. Pandol* and Ling Li**

Supporting Information

Reactive Oxygen Species-Responsive Nanoparticles towards Extracellular Matrix Normalization for Pancreatic Fibrosis Regression

Liang Qi[a,1], Bo-Wen Duan[b,1], Hui Wang[a], Yan-Jun Liu[b], Han Han[b], Meng-Meng Han[b], Lei Xing[b,c], Hu-Lin Jiang[b,c,*], Stephen J. Pandol[d,e,*], Ling Li[a,f,g,*]

[a] Department of Endocrinology, Zhongda Hospital, School of Medicine, Southeast University, Nanjing 210009, China

[b] State Key Laboratory of Natural Medicines, Department of Pharmaceutics, China Pharmaceutical University, Nanjing 210009, China

[c] Jiangsu Key Laboratory of Druggability of Biopharmaceuticals, China Pharmaceutical University, Nanjing 210009, China

[d] Division of Gastroenterology, Department of Medicine, Cedars-Sinai Medical Center, Los Angeles, CA 90048, USA

[e] Basic and Translational Pancreatic Research, Cedars-Sinai Medical Center, Los Angeles, CA 90048, USA

[f] Institute of Glucose and Lipid Metabolism, Southeast University, Nanjing 210009, China

[g] Department of Clinical Science and Research, Zhongda Hospital, School of Medicine, Southeast University, Nanjing 210009, China

[1] These authors contributed equally to this work

[*] Corresponding authors

2.2 Experimental Methods

2.2.1 Synthesis and Characterizations of micelles

Synthesis of ROS Response Nanocarrier Materials

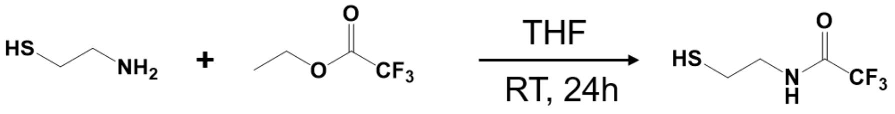

Briefly, 2-aminoethane-1-thiol hydrochloride (2 g, 17.7 mmol), trifluoroacetic anhydride (TFAA) (11.15 g, 53.1 mmol), triethylamine (TEA) (7.36 mL, 53.1 mmol) and tetrahydrofuran (THF) (50 mL) were added into a 100 mL round-bottom flask and kept stirring at room temperature under nitrogen protection for 24 h (TLC, hexane: ethyl acetate = 3:1, v/v). The crude was purified by column chromatography (hexane: ethyl acetate =10:1 ~ 5:1, v/v) to obtain 2.5 g colorless transparent liquid (named R1, yield 81.6%). ^{1}H-NMR (300 MHz, CDCl3), δ(ppm): 3.62 (m, 2H, NHCH2), 2.87-2.90 (m, 2H, SCH2).

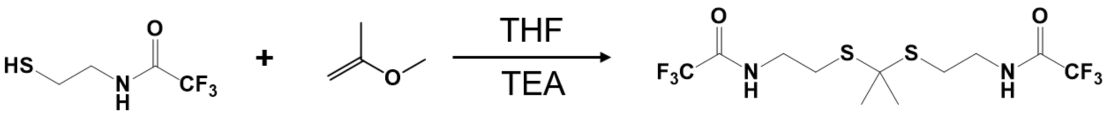

R1 (2.5 g, 14.4 mmol), 2-methoxyprop-1-ene (0.50 g, 7.0 mmol), 4-methylbenzene sulfonic acid (TsOH) (0.25 g, 1.44 mmol) and THF (20 mL) were added into a 50 mL round-bottomed flask and kept stirring at room temperature under nitrogen protection for 48 h (TLC, hexane: ethyl acetate = 3:1). The crude was purified by column chromatography (hexane: ethyl acetate = 10:1 ~ 5:1) to obtain 2.2 g colorless transparent liquid (named R2, yield 78.9%). ^{1}H-NMR (300 MHz, CDCl3),

δ (ppm): 6.87 (s, 2H, 2NH), 3.62-3.64 (m, 4H, NHCH2), 2.87-2.90 (m, 4H, SCH2), 1.67 (s, 6H, 2CH3).

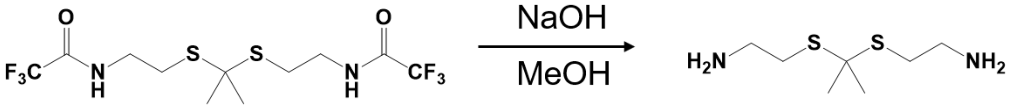

R2 (2.2 g, 5.9 mmol) was added to a 50 mL round-bottom flask and then sodium hydroxide (2 M, 20 mL) was added dropwise and kept stirring at room temperature for 6 h (hexane: ethyl acetate = 1:1, v/v). The crude was extracted with ethyl acetate (3 × 20 mL). The ethyl acetate layer was combined and washed with saturated brine (2 × 30 mL) and then dried overnight with anhydrous sodium sulfate. After filtration and evaporation, 0.8 g light yellow oil was obtained (R3, yield 68.8 %) and directly added to the next reaction without further purification.

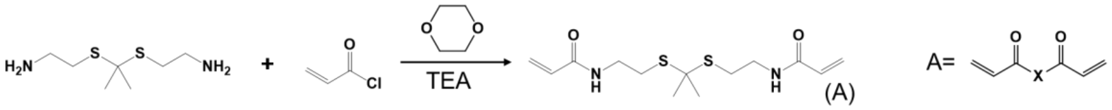

R3 (0.8 g, 4.0 mmol) and TEA (0.810 g, 8.0 mmol) were added to a 50 mL round-bottom flask, and then acryloyl chloride (0.724 g, 8.0 mmol) was added dropwise and kept stirring at ice bath for 0.5 h (TLC, dichloromethane: methanol = 10:1). The crude was purified by column chromatography (hexane: ethyl acetate =1, v/v) to obtain 0.8 g colorless transparent liquid (named R4, yield 66.5%).

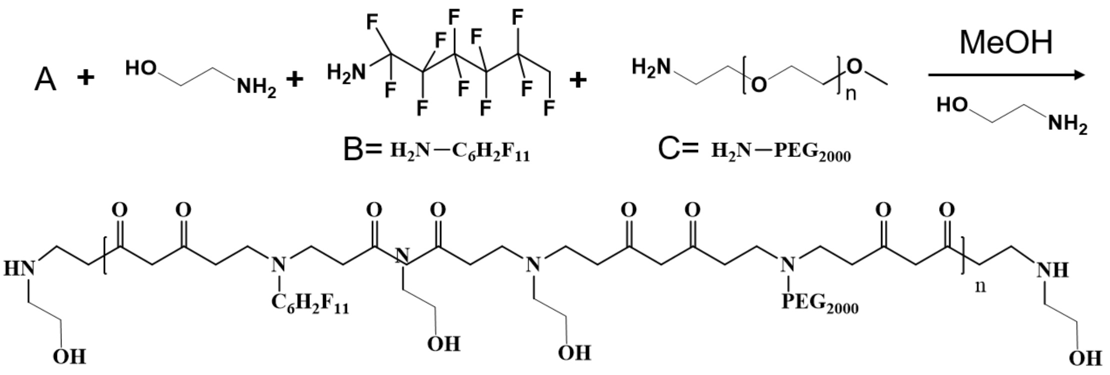

R4 (0.8 g, 2.65 mmol), cholamine (0.028 g, 0.265 mmol), undecylfluorhexylamine (0.079 g, 0.265 mmol), NH2-PEG2000 (0.53 g, 0.265 mmol) and methanol (20 mL) were added to a 50 mL round-bottom flask and kept stirring at room temperature for 72 h. The crude was precipitated with ether dialyzed with water in a dialysis bag (MWCO = 3500 Da) for 2 days. After dialysis, the liquid was freeze-dried to obtain yellow bulk solid (named R5).

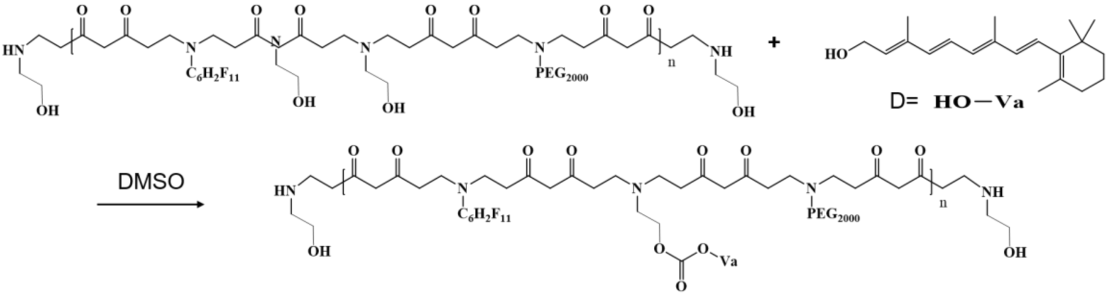

R5 (0.5 g), retinol (0.028 g, 0.265 mmol), N, N`-carbonyldiimidazole (0.086 g, 0.53 mmol) and DMSO (5 mL) were added to a 25 mL round-bottom flask and kept stirring at room temperature for 24 h. The crude was precipitated with ether dialyzed with water in a dialysis bag (MWCO = 3500 Da) for 2 days. After dialysis, the liquid was freeze-dried to obtain yellow bulk solid (named R6).

Preparation of LR-SSVA

LR-SSVA was synthesized by self-assembly and charge adsorption methods. The nanocarrier SSVA and RES were dissolved in 50 μL DMSO; the mixture was added into 2 mL of normal saline drop by drop, and stirred overnight at RT to obtain slightly milky micelle solution. Subsequently, siLoxl1 was dissolved in DEPC water, with the volume ratio of DEPC water and micelle solution 1:1, and then LR-SSVA was obtained after vortex for 30 s. C6-SSVA and siC6-SSVA used hereinafter were prepared in the same way.

Characterizations of LR-SSVA

The size distribution, polymer dispersity index (PDI) and zeta potential were measured by Zetasizer Nano (ZS90, Malvern, UK) for 10 runs. Transmission electron microscope (TEM) system (H-7650, Hitachi, Tokyo, Japan) was applied for the micromorphology of various micelles.

ROS was detected by DCFH-DA method. mPSCs were cultured in a 6-well plate with a density of 8×10^4 cells per well for 24 h. After washed by PBS for 3 times, PBS, LR-SVA (non-ROS response) and LR-SSVA were added into the plate for another 24 h. Subsequently, the culture medium was removed and 1 mL of DCFH-DA was added to each well and incubated at 37℃ for 30 min to monitor the green fluorescence intensity at 488 nm.

Assay of Loading Ability

The content of RES was monitored by detecting the absorbance at 468 nm with Multiskan GO (Thermo Fisher Scientific, USA). The encapsulation efficiency (EE)

and the drug loading efficiency (DLE) of the LR-SSVA were calculated referring to Equation (1) and (2), respectively.

$$EE\ (\%) = \frac{mass\ of\ RES\ encapsulated\ in\ the\ micelles}{mass\ of\ RES\ innicially\ added} \times 100\% \tag{1}$$

$$LE\ (\%) = \frac{mass\ of\ RES\ encapsulated\ in\ the\ micelles}{total\ mass\ of\ materials\ and\ drugs\ weighed\ after\ lyophilization} \times 100\% \tag{2}$$

Stability of Nanoparticles

The stability of LR-SSVA was determined by measuring the change of particle size with time (6, 12, 24, 36, 48, 60 and 72 h) in various buffers. Briefly, LR-SSVA was suspended in saline, phosphate buffered solution (PBS) and 1 mL DME/F12 with 10% fetal bovine serum (FBS), respectively, and the particle size of them were measured at the specified time point.

Assay of Drug Response Release

2 ml of LR-SSVA solution was put into the pre-treated dialysis bag (3500D), accordingly, 20 mL of PBS with 1% Tween 80 (v/v) was added into the Erlenmeyer flask, and shaken at 37°C with the rate of 120 rpm. One group was added with hydrogen peroxide solution (20 mM), and the other with equal quality of PBS for negative control. Samples were taken at 0 min, 30 min, 1 h, 2 h, 4 h, 6 h, 8 h, 12 h, 24 h, 48 h, 72 h and 96 h, respectively, for the detection of RES concentration.

Gene Binding Capacity and Degradation Resistance of LR-SSVA

The siRNA binding capacity of SSVA was verified by agarose gel

electrophoresis, and the electrophoresis conditions were 100 V, 15 min and 0.5×Tris/Borate/EDTA buffer solution. Specifically, 0.5 g of agarose powder was put into a conical flask, and was heat to dissolve evenly in a microwave oven. The agarose solution was poured into the glue mold after cooling to 50-60°C, and then a comb is inserted in the appropriate position. After the gel was solidified, it was transferred to an electrophoresis tank, and samples with different mass ratios (SSVA/siRNA, 0.5~20) were carefully added to the sample tank. When the band moved to about 2 cm from the front of the gel, electrophoresis was stopped for Observation of images under the gel imaging system.

Gel electrophoresis was applied to evaluate the protective ability of SSVA to siRNA, and the electrophoresis conditions were 100 V, 25 min and 0.5×Tris/Borate/EDTA buffer solution. Specifically, the sample with mass ratio of 10: 1 was prepared (siRNA, 0.2 μg), and the RNase A/Mg2+ and/or heparin sodium (5%) were added to incubate at RT for 2 h according to different grouping. After electrophoresis, the images were observed under the gel imaging system.

2.2.2 Cells and Cellular Pharmacodynamics

Cell Culture

The Dulbecco's modified Eagle's medium (DMEM)/Ham's F12 (1:1 v/v, Gibco, USA) was supplemented with 10% (v/v) FBS (Thermo Fisher Scientific, Waltham, Massachusetts, USA) and 1% penicillin and strep- tomycin (Gibco, Grand Island, USA) for culturing the pancreatic stellate cells (PSCs) lines of human (hPSCs) and

mouse (mPSCs). The mouse pancreatic acinar carcinoma cell line (266-6) was purchased from Zhongqiao Xinzhou Biotechnology Co., Ltd. (Shanghai, China), and cultured in DMEM media (Zhongqiao Xinzhou, ZQ-100) containing 10% (v/v) FBS and 1% penicillin and streptomycin. Cells were all cultured with 5% carbon dioxide (CO_2) atmosphere at 37°C.

Cytotoxicity Assay

Firstly, a density of 1×10^4 cells per well of 266-6 cells and PSCs were seeded in 96-well plates to incubate for 24h, after which the growth medium was replaced by 100 μL of two vehicles with/without RES loaded at different concentrations. Then, 20 μL of MTT (5mg mL^{-1}) was added in each well after further incubation for 24 h, which should be replaced by DMSO after incubating at 37 °C for 4 h. Finally, microplate reader (Thermo Fisher Scientific, USA) was applied to detect the absorbance of each well at 570 nm. Experiments were all conducted with three secondary wells.

Cellular Uptake and Lysosome Escape

Coumarin 6 (C6) was regarded as fluorescent probe for the investigation of *In vitro* uptake of micelles. C6-loaded micelles were synthesized as previous description. 1×10^5 cells per dish of mPSCs were seeded in glass-bottomed petri dish to incubate overnight. Thereafter, serum-free medium containing C6, C6-S and C6-SSVA was added and the cells continued to incubate for 2 h to 4 h. DAPI was used to stain cell nucleus after washed by PBS, and the images were obtained by Confocal laser

scanning microscopy (CLSM) (Olympus, Tokyo, Japan). Quantitative analysis of cellular uptake was conducted with cell suspension after digestion, by detecting fluorescence intensity of C6 with flow cytometry.

In order to explore the uptake mechanism of LR-SSVA, the uptake inhibitors genistein (5 μg/mL), chlorpromazine (10 μg/mL) and amiloride (50 μM) were incubated with cells for 1 h in advance, respectively. The cells were washed with PBS and digested with trypsin to harvest the cell suspension. After centrifuged (2000 rpm, 5 min), the supernatant was discarded, and the cells were resuspended in 400 μL PBS for detection of C6 fluorescence intensity by flow cytometry.

Additionally, lysosome escape assay was conducted by siFAM-adsorbed micelles. The cells were treated with siFAMR-SSVA for 3 h to 6 h, and lysosome was stained by Lyso Tracker Red DND. CLSM was used to obtain the images and to analyze co-localization.

Immunofluorescence Staining

mPSCs were washed by PBS and fixed by 4% paraformaldehyde after treating with different preparations. Hank's balanced salt solution (HBSS) was used to wash cells and 5% donkey serum was applied for blocking. Subsequently, rabbit anti-human α- alpha smooth muscle actin (α-SMA) primary antibody (Abcam, Cambridge, UK) was added and incubated 4°C. After which, cells were incubated with AF657-conjugated goat anti-rabbit secondary antibody (Beyotime Biotechnology, Shanghai, China), and DAPI dye was used to counterstain the nuclei. At last, CLSM

was used to assess cell fluorescence and the images were quantified by Image J software.

Assay of Cell Invasion and Migration

Transwell device was coated with rat tail collagen I (0.3 mg mL-1, 50 μL) in advance; 500 μL of mPSCs in FBS-free medium were seeded onto the collagen layer, and equal growth medium was simultaneously added into the 24-well plates. After co-incubating with various preparations for 24 h, the cells in the upper layer were gently scraped, while those outside the chamber were fixed with 4% paraformaldehyde for further staining by 0.1% crystal violet. Inverted fluorescence microscope was applied for imaging, and Image J software was used for semi-quantification.

mPSCs were seeded onto the upper chamber and cultured for 24 h, after which the cells were treated with various preparations to incubate another one day. The transepithelial electrical resistance (TEER) was determined by recording the resultant resistance after inserting the TEER probe into the apical and basal compartment of the transwell. TEER was recorded before and after treatments to assess the migration ability.

2.2.3 Animals and Pharmacodynamics In vivo

Animal Models

Male C57BL/6J mice of six-week-old were purchased from Vital River Laboratory Animal Technology Co., Ltd. (Beijing, China). All of the animal

experiments were conducted under the protocols approved by the Ministry of Health of the People's Republic of China and followed the Guidelines for the Care and Use of Laboratory Animals of China Pharmaceutical University. Mice were raised with 12 h light/dark cycle at 25°C, and fed for 1 week with water and standard laboratory chow before experiments. There were 123 mice selected randomly for modeling, and 10 mice served as normal controls. Caerulein (Bachem, Bubendorf, Switzerland) was diluted in normal saline, and mice were intraperitoneal (ip) injected for 6 weeks to establish CP model (50 μg kg^{-1}, 6 hourly injections/day, 3 days/week). The same volume of normal saline was used for control group with the same method.

Acute and Short-term Toxicity Studies

Six healthy mice were divided into 2 groups. One group was given LR-SSVA at three times of the therapeutic dose for three days, and the other group was given PBS as negative control. Blood samples and main organs (heart, liver, spleen, lung and kidney) were collected for H&E staining and liver/kidney function assessment to evaluate the acute toxicity of micelles.

Additionally, blood and organs (liver, lung, heart, spleen and kidney) were harvested after the final injection of pharmacodynamic studies, for H&E staining and liver/kidney function detecting to evaluate the short-term toxicity of micelles.

Pharmacodynamics and In vivo Imaging Studies

For the pharmacodynamic studies, 81 of the fibrotic mice were assigned into 9 groups from the fourth week, and injected through tail vein with free RES, R-SS,

L-SS, R-SSVA, L-SSVA, LR-SS and LR-SSVA in PBS or PBS alone twice a week. After the final injection, mice were sacrificed to collect blood and organs (pancreas, liver, lung, heart, spleen and kidney) for further experimentation.

For the living imaging studies, 21 of fibrotic mice were divided into 3 groups, and DiR was chosen as fluorescent probes. Each group of mice were injected with free DiR, DiR-SS and DiR-SSVA (DiR dose, 2 mg kg^{-1} body weight), respectively; afterwards the fluorescence at various time points (3 h, 6 h, 12 h, 24 h and 48 h) was imaged with excitation and emission wavelengths of 745 nm and 800 nm, by In vivo imaging system (FX-Pro; Bruker).

For the localization analysis, 21 of fibrotic mice were included for 3 groups. Free DiI, DiI-SS and DiI-SSVA (DiI dose, 2 mg kg^{-1} body weight) were injected via tail vein for three consecutive days. After the last injection, mice were sacrificed to harvest pancreas for frozen sections. Rabbit anti-mouse antibody of α-SMA and DAPI dye were used for section staining, and the co-localization of α-SMA and DiI was visualized by CLSM.

Serological Indicators

Serum levels of blood urea nitrogen (BUN), creatinine (Cr), alanine aminotransferase (ALT), aspartate aminotransferase (AST) and serum amylase (AMY) activity were determined by automatic biochemical analyzer in Servicebio Biotechnology Co., Ltd. (Wuhan, China). Hydroxyproline levels in pancreas, serum SOD, MDA and GSH-PX were tested by standard assay kit following the

manufacturer's instructions.

The serum and pancreatic concentrations of transforming growth factor-β (TGF-β), interleukin-1β (IL-1β), interleukin-6 (IL-6), tumor necrosis factor-α (TNF-α) and LOXL1 were detected by enzyme-linked immunosorbent assay (ELISA) kit against their specifications.

Histological Analysis

Pancreas tissues were embedded by paraffin, and sectioned for staining. H&E staining Kit, Masson's trichrome staining Kit, and and 0.1% (w/v) Sirius Red were applied in accordance with the specifications. Immunohistochemistry was carried out referred to the standard protocols, during which the rabbit anti-mouse collagen I, a-SMA, fibronectin and LOXL1 primary antibody and goat anti-rabbit secondary antibody were used for stanning. Primary antibodies of collagen I, a-SMA and fibronectin were purchased from Abcam (Cambridge, UK), LOXL1 was from Novus (USA), and secondary antibody was obtained from Servicebio Biotechnology Co., Ltd.

Dihydroethidium (DHE) can freely get into living cells and be oxidized by intracellular ROS to form ethidium oxide, which can be mixed into chromosome DNA to produce red fluorescence and reflect ROS content.

Scanning Electron Microscope (SEM)

The pancreas samples of different groups were cut into small pieces and decellularized for 4-12h, in PBS with 0.1% penicillin/streptomycin (Sigma-Aldrich)

and 1% sodium dodecyl sulfate (Sigma-Aldrich). Later on, the tissues were washed with distilled water, lyophilized and sputter-coated with gold for observation under a JSM-IT200LA (JEOL, Tokyo, Japan).

Western Blotting

Protein from cells and pancreas tissues was extracted according to the standard procedures, and was transferred to polyvinylidene difluoride (PVDF) membranes after isolation by sodium dodecyl-polyacrylamide gel electrophoresis (SDS-PAGE). After blocking in 5% skim milk for 2 h at RT, membranes were incubated with primary antibodies (collagen I, a-SMA, fibronectin, LOXL1) and β-actin rabbit anti-mouse antibodies) at 4°C overnight, and followed with secondary antibodies at RT for 2 h. Protein bands were visualized by CCD image system (Tanon 4200, Shanghai, China), and Image J software was used for semi-quantitative analysis.

Table S1. Characteristics of different preparations.

SSVA:RES, w: w	EE (RES, %)	DLE (RES, %)
7.5/0.1	91.94±0.72	1.21±0.01
7.5/0.25	86.95±1.04	2.83±0.04
7.5/0.5	83.67±1.25	5.77±0.62
7.5/0.75	72.45±3.87	6.73±0.38

Table S2. Characteristics of different preparations.

	EE (RES, %)	DLE (RES, %)
R-SS	84.83±1.12	5.86±0.24
R-SSVA	84.33±0.92	5.81±0.13
LR-SS	83.29±0.90	5.64±0.03
LR-SSVA	83.67±1.25	5.77±0.62

Table S3. Primers used for qPCR.

Gene	Forward Primer	Reverse Primer
gapdh	ATGGTGAAGGTCGGTGTGAAC	GCCGTGAGTGGAGTCATACTG
loxl1	CGCUACGUUUCUACAACAATT	UUGUUGUAGAAACGUAGCGTT

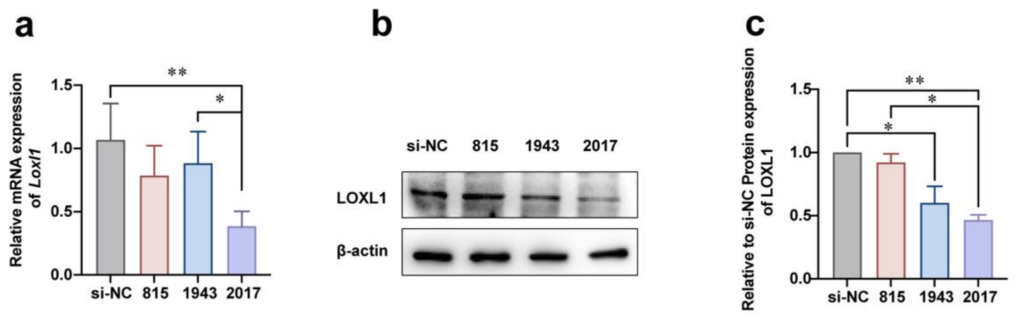

Fig. S1 The inhibition efficiency of siLOXL1 interference fragment evaluated by qPCR (a) and Western blot (b, c). Values are expressed as means ± SD (*P < 0.05, **P < 0.01; n = 3).

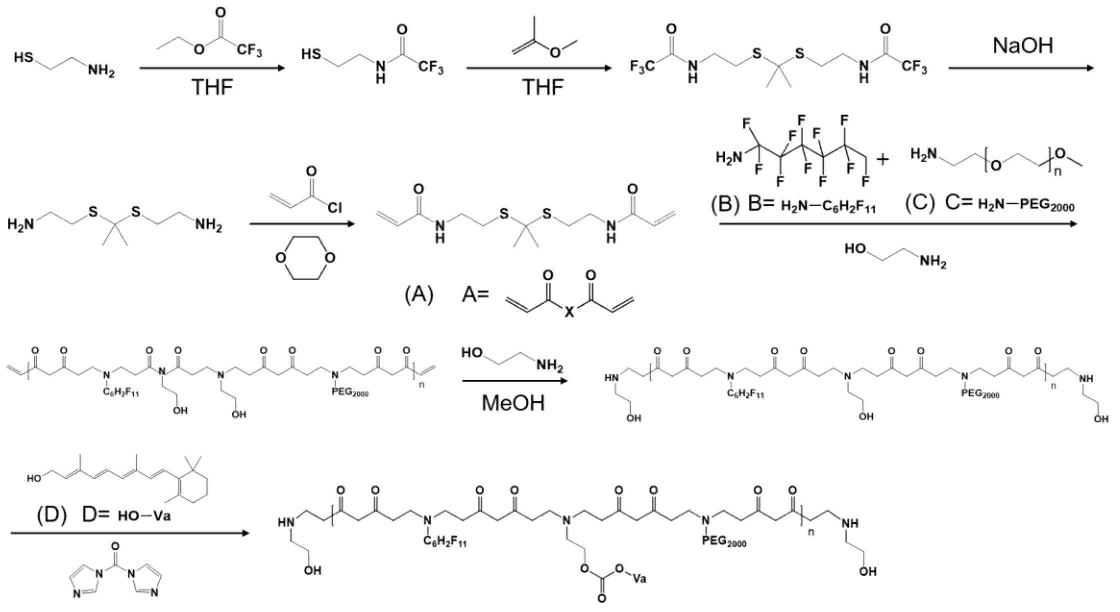

Fig. S2 The synthetic process of LR-SSVA.

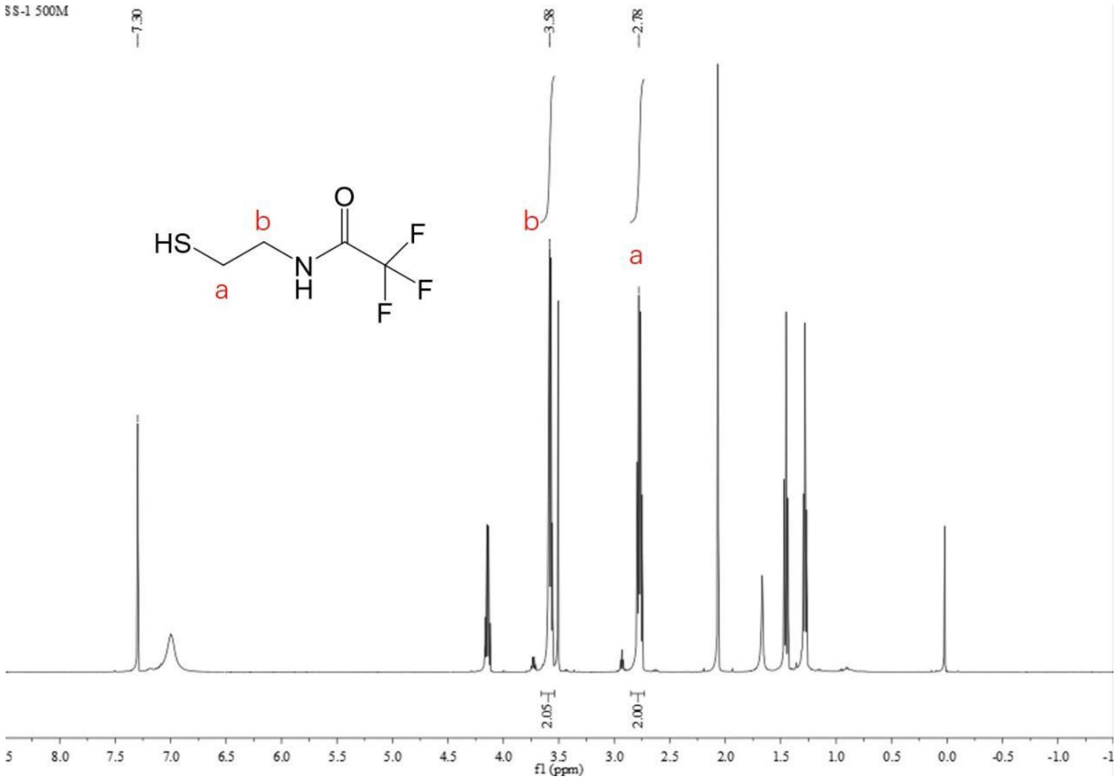

Fig. S3 ^{1}H-NMR spectra of R1.

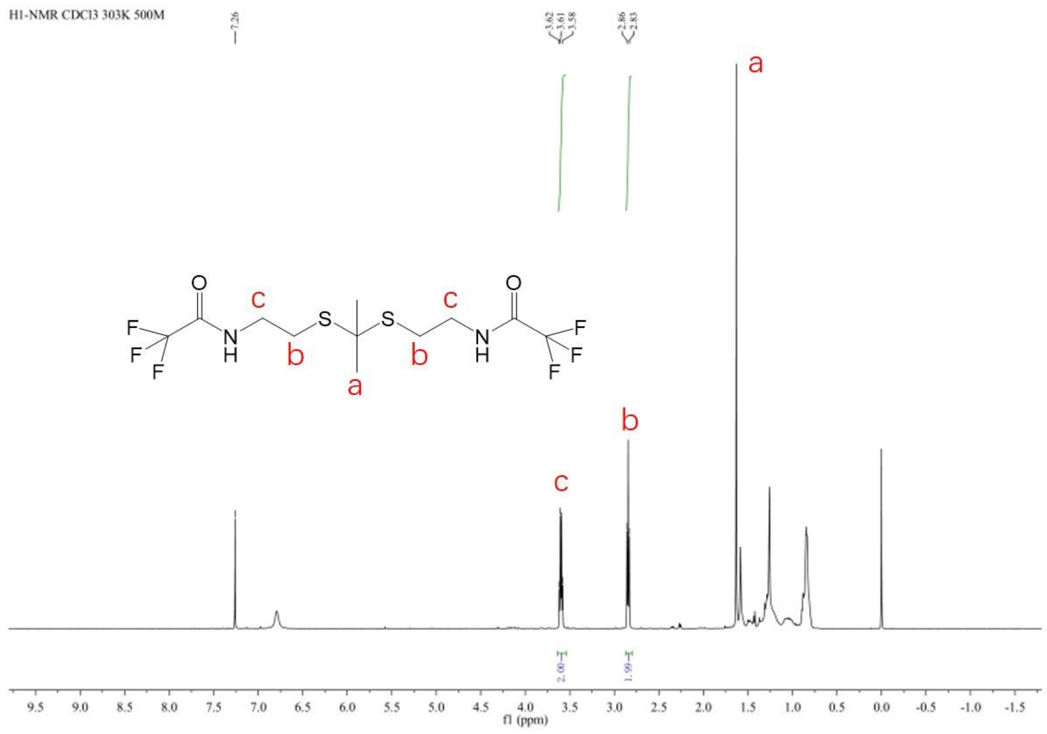

Fig. S4 ^{1}H-NMR spectra of R2.

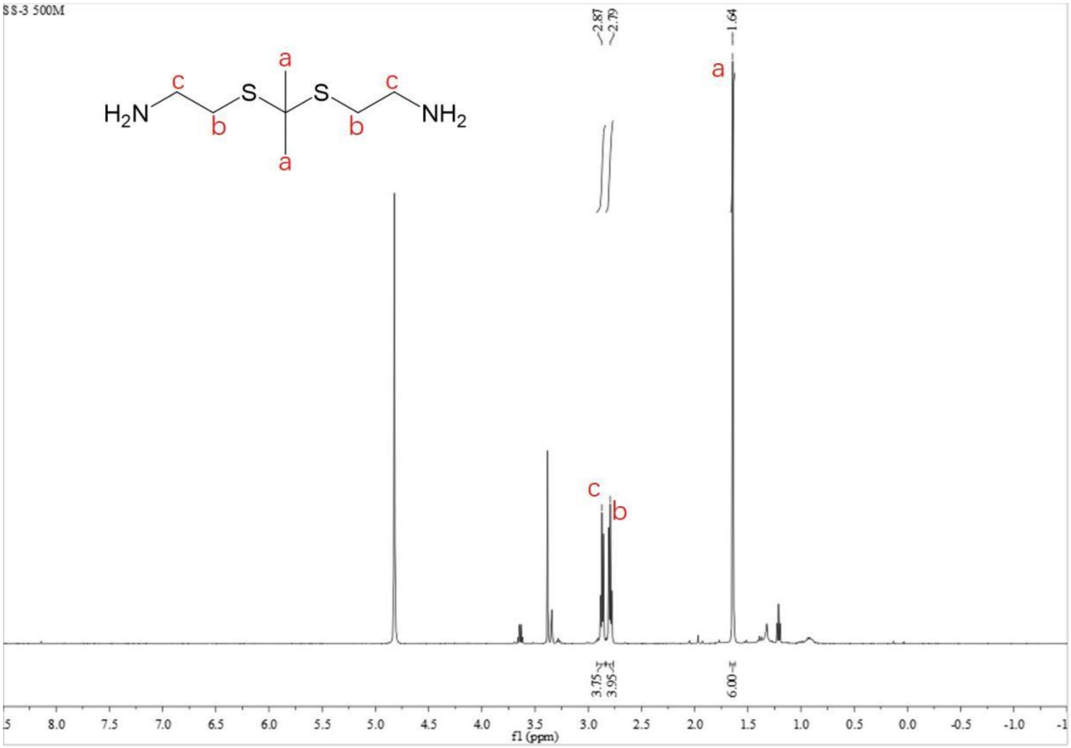

Fig. S5 ^{1}H-NMR spectra of R3.

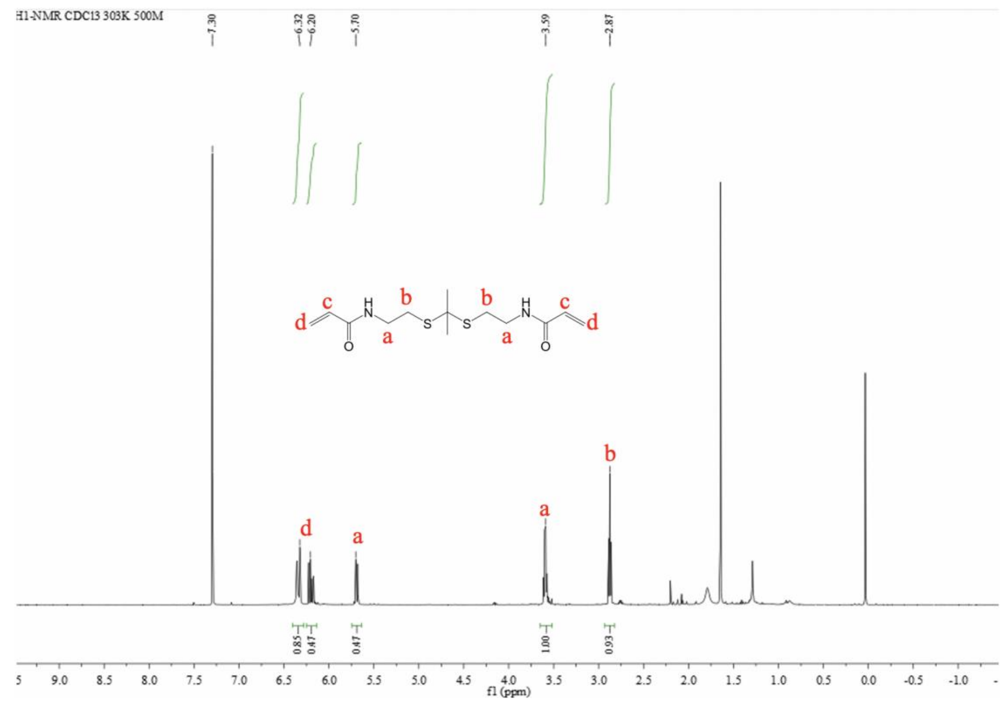

Fig. S6 ^{1}H-NMR spectra of R4.

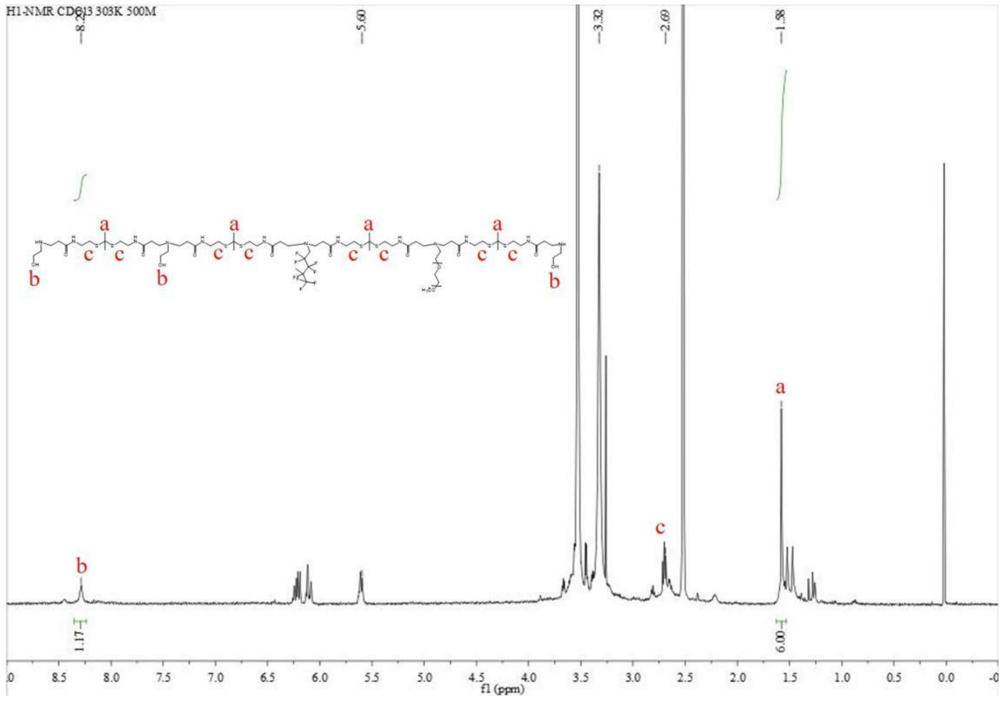

Fig. S7 ^{1}H-NMR spectra of R5.

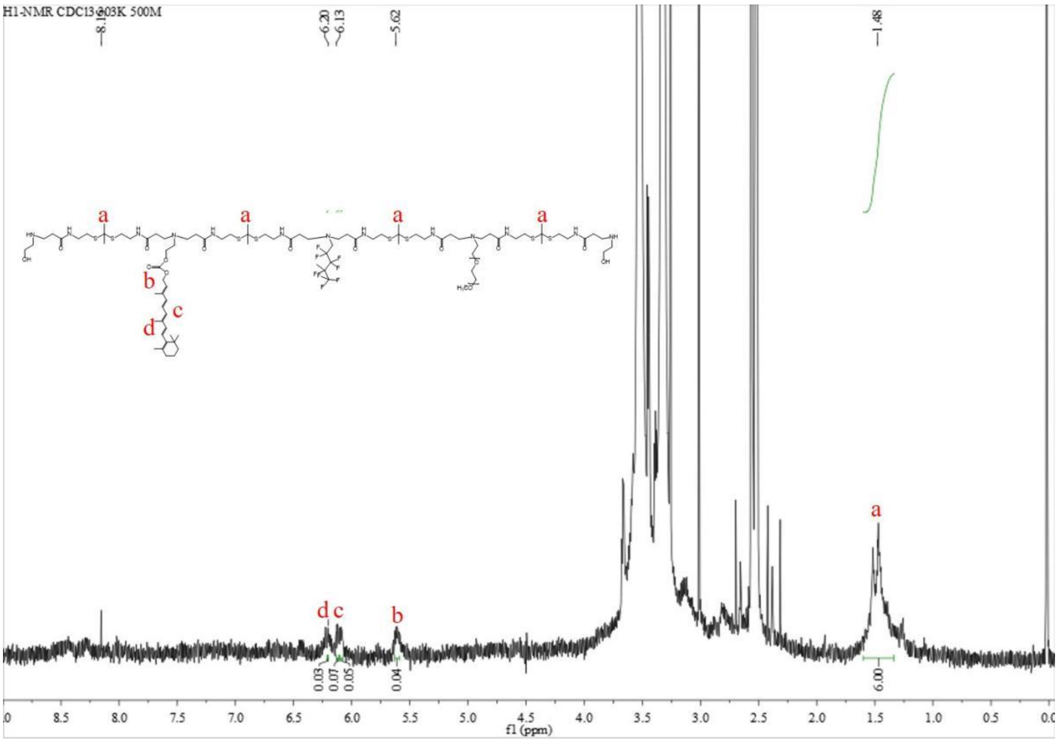

Fig. S8 ^{1}H-NMR spectra of R6.

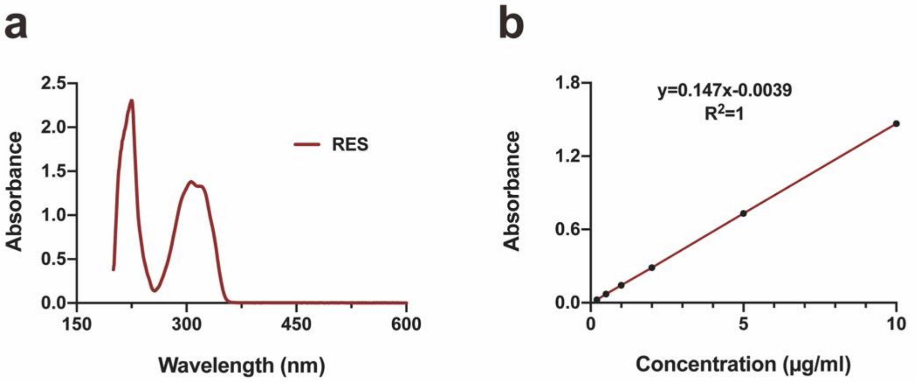

Fig. S9 (a) Ultraviolet-visible spectra of RES. (b) Standard curve of RES.

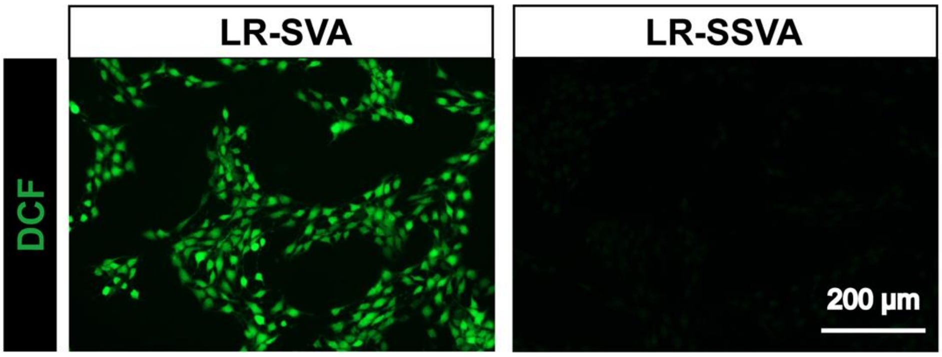

Fig. S10 Detection of cellular ROS by DCFH-DA method. Green fluorescence represents ROS accumulation.

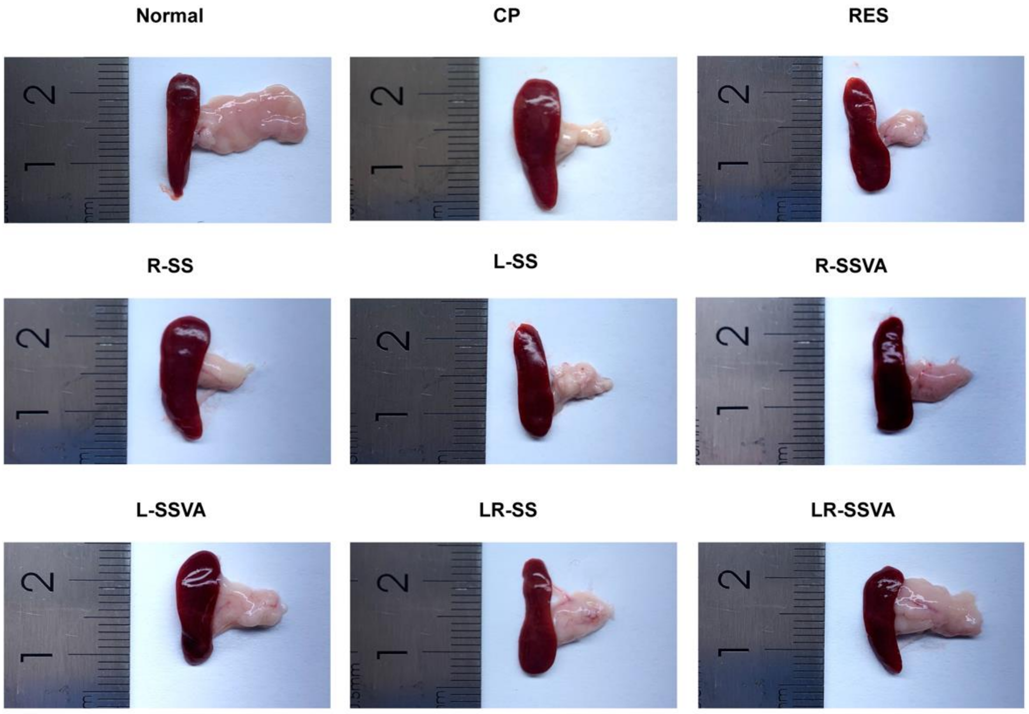

Fig. S11 The images of the general changes of pancreas in the control group, the caerulein modeling group and other groups after various treatments. [Photographed by Liang Qi]

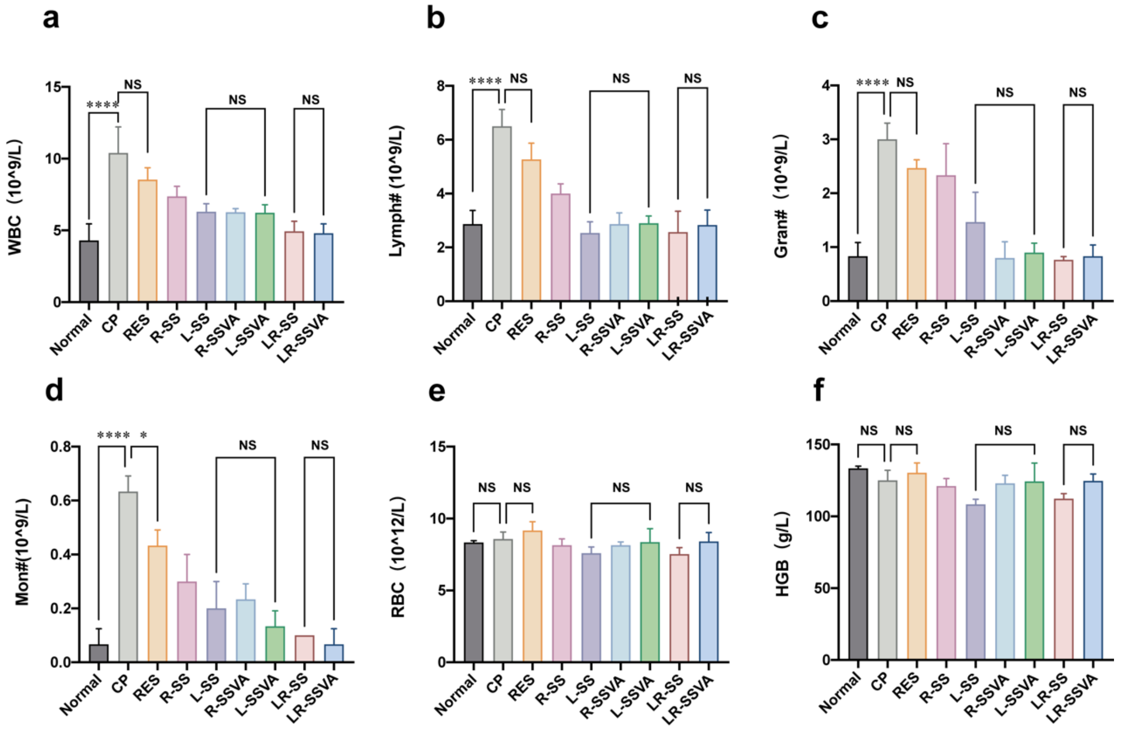

Fig. S12 The number of (a) white blood cell (WBC), (b) lymphocyte (Lymph#), (c) neutrophils (granulocyte #), (d) monocyte (Mon#), (e) red blood cell (RBC) and (f) hemoglobin (HGB) in the whole blood of mice.

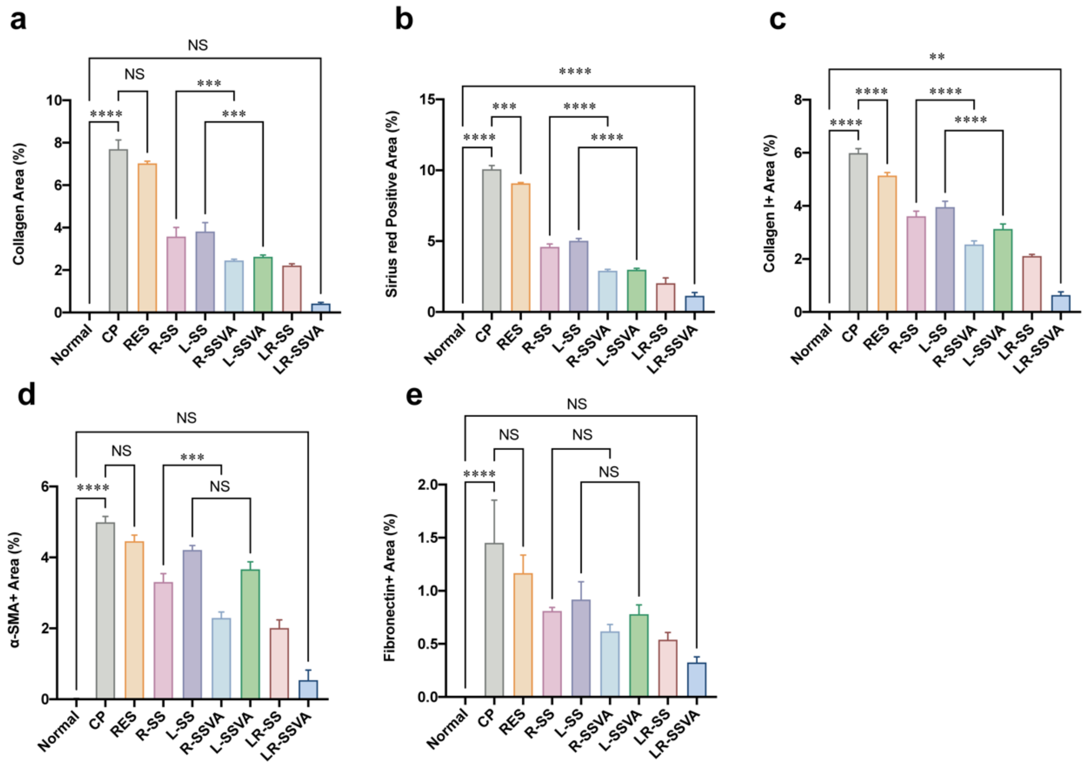

Fig. S13 Semi-quantitative results of Masson (a), Sirius Red (b) and IHC staining (c-e) of pancreas tissue sections. Values are expressed as means ± SD (**P < 0.01, ***P < 0.001, ****P < 0.0001, NS, no significant difference).

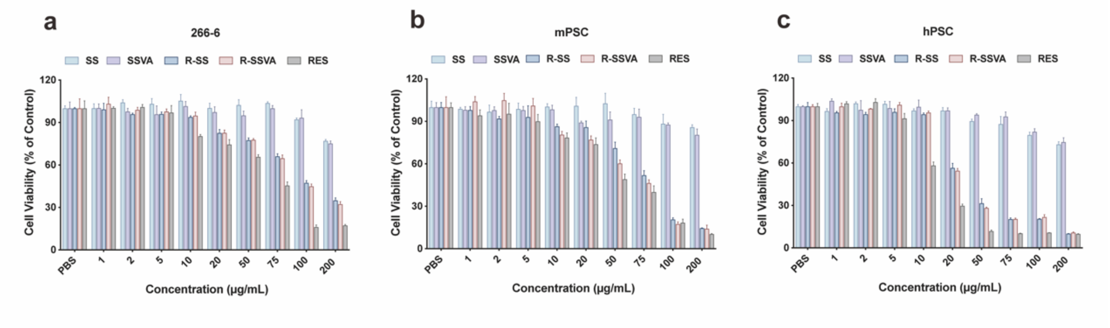

Fig. S14 Cytotoxicity of SS, SSVA, R-SS, R-SSVA, and free RES in (a) mPSCs, (b) hPSCs and (c) 266-6 cells at 24 h, respectively.

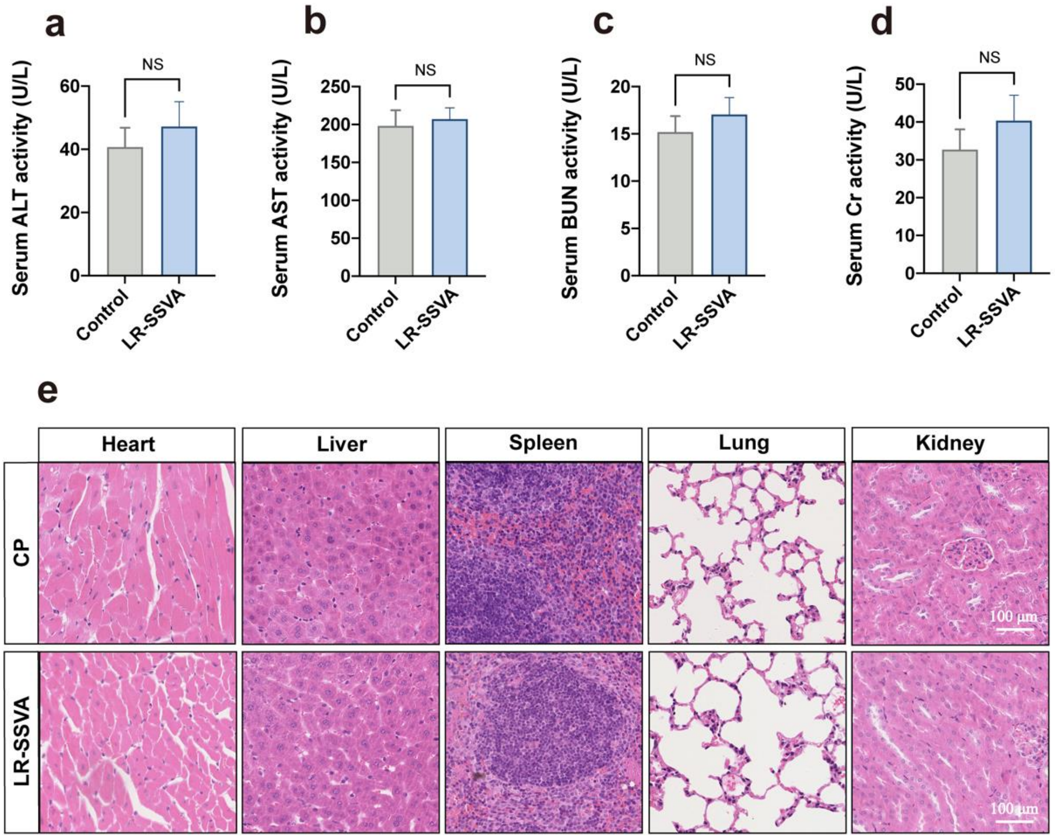

Fig. S15 Acute toxicity *In vivo* of LR-SSVA. (a-d) Serum activities of ALT, AST, BUN and Cr, as measured by biochemical assays. (e) Representative of H&E staining

of heart, liver, spleen, lung and kidney tissue sections from mice after treated with LR-SSVA. Scale bar: 100 μm. 200×. Values are expressed as means ± SD (NS, no significant difference; n = 3).

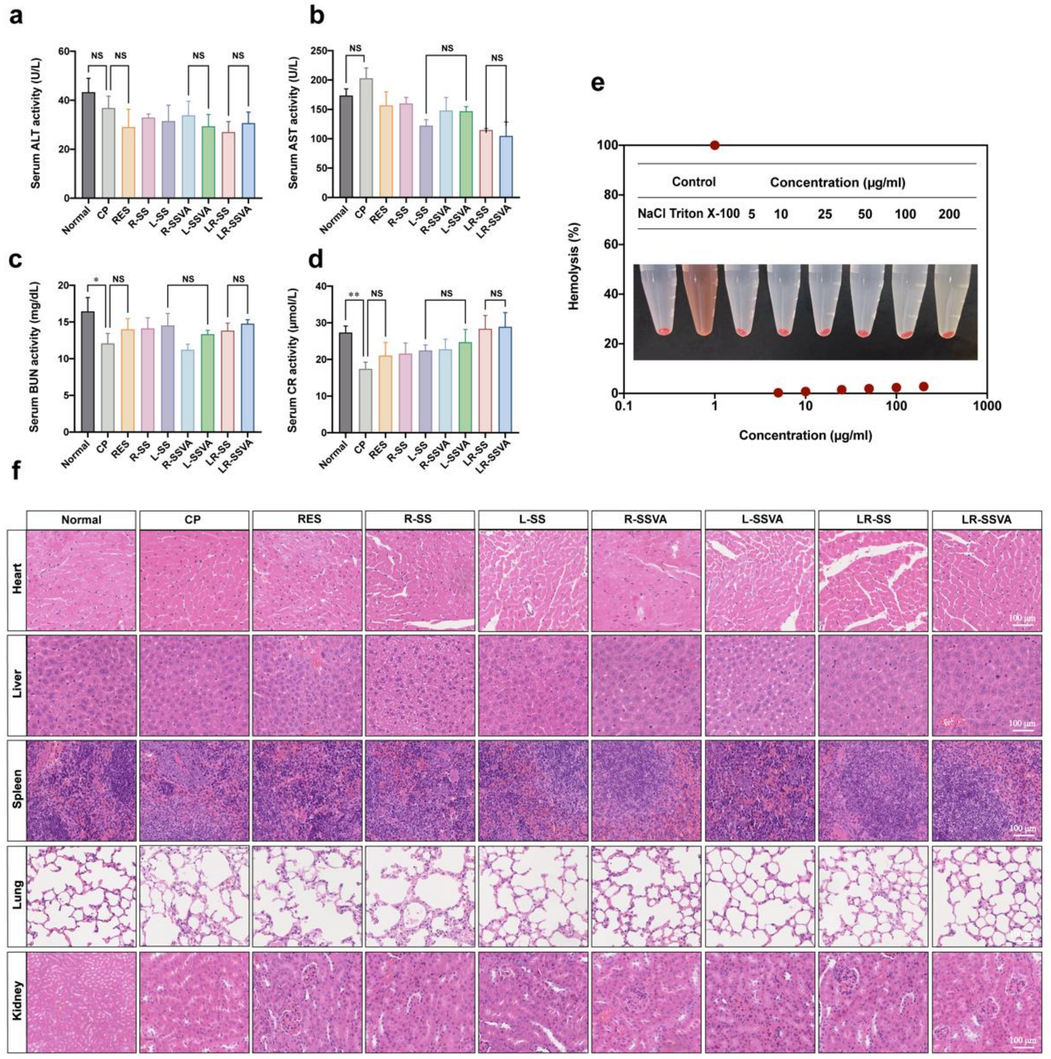

Fig. S16 Short-term toxicity *In vivo* of LR-SSVA over the experimental period. (a-d) Serum activities of ALT, AST, BUN and Cr, as measured by biochemical assays. (e) Visual observation of hemolysis caused by siRES/SSVA in PBS at pH 7.4. (f) Representative of H&E staining of heart, liver, spleen, lung and kidney tissue sections from mice after different treatments. Scale bar: 100 μm. 200×. Values are expressed as means ± SD (*P < 0.05, **P < 0.01, NS, no significant difference; n = 3).

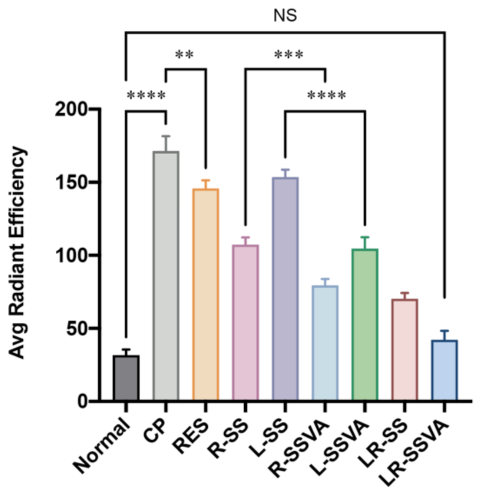

Fig. S17 Semi-quantitative results of DHE staining of ROS in healthy and PF mice after different treatments. Values are expressed as means ± SD (**P < 0.01, ***P < 0.001, ****P < 0.0001, NS, no significant difference).
